# Recycling of animal protein wastes in the formulation of feed for *Labeo rohita* and *Mystus vittatus*—a comparative evaluation

**DOI:** 10.1007/s11250-024-03910-6

**Published:** 2024-03-02

**Authors:** Ayan Samaddar, Anilava Kaviraj, Izabela Nielsen, Subrata Saha

**Affiliations:** 1 WorldFish - India, Directorate of Fisheries, Cuttack, 753001 Odisha India; 2 Department of Zoology, University of Kalyani, Kalyani, 741235 West Bengal India; 3 Department of Materials and Production, Aalborg University, Aalborg, 9220 Denmark; 4 Department of Mathematics, University of Engineering & Management, Kolkata, 700160 India

**Keywords:** Fermentation, Fish-meal, Amino acid, Hyperaminoacidemia, Optimum replacement

## Abstract

Lactic acid bacteria (LAB) are key players in the fermentation of organic wastes and their recycling as feedstuff for fish. Whey, a common dairy byproduct in India, is a cheap source of LAB and can be used to ferment animal byproducts. An experimental study was designed to explore whether the whey fermented animal protein blend (WFAPB) could be used as a fishmeal replacer in the formulation of feed for both stomach-less carp fish *Labeo rohita* and stomach-bearing catfish *Mystus vittatus*. Experiments were performed with five isoproteinous, isolipidous, and isoenergetic feeds with WFAPB replacing fishmeal (FM) by 0% (T1), 25% (T2), 50% (T3), 75% (T4), and 100% (T5). Fifteen days of laboratory experiments with these experimental feeds revealed that more than 50% FM replacement level could result in excess postprandial absorption (6 h) of some essential and non-essential amino acids in the plasma of both fish. The postprandial absorption was more in *M. vittatus* than *L. rohita*. Ninety-day experiments were conducted in outdoor cement vats to measure growths and deposition of amino acids (AA) in muscle. Regression analysis was performed to find the optimal FM replacement based on four growth parameters and fifteen AA deposition in muscle. A two-phase fuzzy methodology was used to obtain Pareto-optimal replacement levels for each fish. The results demonstrated that FM replacement levels were 7.63% and 36.79% respectively for *L. rohita* and *M. vittatus* when only four growth parameters were considered. However, based on the FM replacement level that maximized deposition of 15 amino acids and growth parameters, it was found that 12.23% and 40.02% replacement of FM by the WFAPB was ideal respectively for *L. rohita* and *M. vittatus*. The results revealed that only a fraction of both essential and non-essential amino acids absorbed in plasma could be converted into protein and deposited as bound amino acids in the muscle. It is concluded that fermentation by whey is an inexpensive, easily available, and environmentally sustainable technique to recycle animal protein in the formulation of feed for fish, and the stomach-bearing carnivorous fish are more efficient in utilizing fermented animal protein blend than the stomach-less carps.

## Introduction

Protein turnover, the balance between protein synthesis and protein degradation, is considered to be the central issue that controls the growth of fish (Katersky and Carter, 2010; Carter et al., 2012; Matias et al., 2023; Youssef et al., 2023). It reflects how closely dietary protein matches quantitative and qualitative requirements of amino acids (AA) by fish (Panserat and Kaushik, 2010; Mente et al., 2021). Growth in fish is linearly correlated with intake of adequate and balanced amounts of both essential and non-essential amino acids (Katersky and Carter, 2010; Kaushik and Seiliez, 2010; Teles et al., 2020; Dileep et al., 2021). Fishmeal (FM) serves as an ideal protein source in the formulation of feed for fish because it contains all the essential amino acids (EAA) in balanced proportion and has an excellent turnover value (Tacon and Metian, 2015; Hua et al., 2019; Mugwanya et al., 2023; Dileep et al., 2021). However, due to the increasing demand and cost of FM, searches for low-cost sustainable FM replacers have been the priority research in recent years (Obirikorang et al., 2015; Naylor et al., 2021; Mbokane et al., 2022; Simtoe et al., 2022; Mugwanya et al., 2023). Fish offal (FO) and slaughterhouse blood are two protein-rich animal wastes that have been widely used in recent years as FM replacers in the formulation of feed for fish (Samaddar et al., 2021). However, all organic wastes need treatments before their use as feed ingredients. Fermentation has been proven to be an efficient technique to process animal by-products before their incorporation in feed formulation (Samaddar et al., 2015; Shabani et al., 2018; Shabani et al., 2021). Fermentation minimizes the loss of nutrients and deactivates the anti-nutritional factors contained in organic wastes (Yu et al., 2019). However, fermentation results in increased availability of free amino acids (FAA), and thus, feed containing fermented products often causes hyperaminoacidemia of some AA in fish.

Hyperaminoacidemia is a phenomenon of a sudden quantitative surge of plasma amino acid levels observed after feeding a FAA enriched diet (Tantikitti and March, 1995; Samaddar et al., 2015). Evidence suggests that hyperaminoacidemic condition leads to imbalances in AA utilization in respect of actual cellular requirements for protein synthesis resulting in lower AA retention (Boirie et al., 1997; Larsen et al., 2012), loss of absorbed AA through oxidation (Brezas and Hardy, 2020), metabolization for energy (Jobling, 2012) leading to reduction in growth of the fish (Espe and Lied, 1994; Stone et al., 1989; Walton et al., 1986; Simtoe et al., 2022; Mugwanya et al., 2023).

The objective of the present study was to explore and compare the efficiency of whey fermented animal protein blend (WFABP) as a FM replacer in the formulation of feed for a herbivorous carp fish *Labeo rohita* and a carnivorous catfish *Mystus vittatus*. Whey is a viable and readily available inoculum to ferment fish-offal wastes and convert them to feed supplement (Samaddar and Kaviraj, 2014; Mayta-Apaza et al., 2022). Samaddar et al. (2015) also observed that WFSHB exhibited a higher amount of FAA than the *Lactobacillus acidophilus* fermented slaughterhouse blood (LFSHB).

Lactic acid bacteria (LAB) are the main players in the fermentation and preservation of organic wastes. Improvisation of fermentation techniques to produce cost-effective, microbiologically safe, and nutritionally sound fermented products suitable for feed ingredients in feed formulation has been given importance in recent years. Whey, a byproduct of the dairy industry, is a potential source of LAB, easily available to the farmers and as efficient as the pure culture of the LAB *Lactobacillus acidophilus* (Samaddar and Kaviraj, 2014; Mayta-Apaza et al., 2022). Therefore, in this study, we compared feed intake rate (FIR), apparent protein digestibility (APD), postprandial (PP) absorption of AA in blood, growth, and AA deposition in muscle between *L. rohita* and *M. vittatus*, both fed WFAPB-supplemented feed. *L. rohita* lacks a true stomach, and digestion begins in the alkaline medium of the intestine affected by the digestive enzymes secreted from the pancreas (Debnath et al., 2007; Goncalves et al., 2016). *M. vittatus*, on the other hand, possesses a true stomach with gastric glands, which initiate the primary digestion of protein with the help of pepsinogen and hydrochloric acid (Chakrabarti and Ghosh, 2014). Pepsinogen is the precursor of pepsin, an enzyme responsible for hydrolyzing proteins in the stomach, and is activated only in the low pH of the stomach. Digestion, however, is completed in the alkaline medium of the intestine, which, unlike the stomach-less fish, *L. rohita* is very short. Because of these morphological and physiological variations, these two species exhibit differences in the digestion of nutrients and the efficiency of their utilization (Samaddar et al. 2015, 2021). Since fermentation results in the simplification of feed ingredients, does this variation matter for their growth and digestive performance when fed a fermented product? A comparative study between the two species is lacking in this respect. In this research, we attempted to evaluate the difference in digestive performance, nutrient utilization, and growth between *L. rohita* and *M. vittatus* fed a feed supplemented by whey fermented animal protein blend. An improvement or comparable performance of the fish in terms of growth and AA deposition in muscle would motivate farmers to use whey as a readily available and cost-effective inoculum to ferment animal protein blend for feed formulation.

**Table 1:** Proximate composition of fishmeal used in the formulation of experimental feeds

Fishmeal (% dry matter basis except gross energy)								
Crude protein	Crude lipid	Crude fiber	Ash	Moisture	NFE	Gross energy (kJ/g)	FAA	
63.16	8.42	3.87	8.63	7.33	8.36	17.72	0.23	
Essential amino acids								
Arg	His	Iso	Leu	Lys	Met	Phe	Thr	Val
4.67	3.44	4.43	5.86	5.16	2.04	2.64	2.38	3.62
Non essential amino acids								
Ala	Asp	Cyst	Glu	Gly	Pro	Ser	Tyr	
3.12	4.18	0.53	5.73	3.11	2.33	1.54	1.98	

Note: Arginine (Arg), Histidine (His), Isoleucine (Iso), Leucine (Leu), Lysine (Lys), Methionine (Met), Phenylalaline (Phe), Threonine (Thr), Valine (Val), Alanine (Ala), Aspartic acid (Asp), Cystine (Cys), Glutamic acid (Glu), Glycine (Gly), Proline (pro), Serine (Ser), and Tyrosine (Tyr)

Aquaculture is one of the fast-growing animal husbandry sectors and involves supplying affordable protein to humans in developing countries (Shamsuzzaman et al., 2020). It is unquestionably revealed that FM as a protein supplement in feeds increases fish production. However, the poor and marginal farmers in remote villages of India, Bangladesh, and the adjoining countries need more financial strength to buy the FM-based prepared feed (Mishra et al., 2022) and thus use either homemade feed or feed with FM replaced by low-cost protein supplements. However, it has yet to be established if such replacement of FM can ensure better outcomes for AA absorption in the blood, growth, or higher deposition of AA in muscle simultaneously. Therefore, considering the feed’s outcome and cost-effectiveness, a trade-off needs to be explored. In this study, we focused on the determination of optimal replacement of FM by WFAPB, which could be based on only weight gain (WG) or a combination of four growth parameters (WG, SGR FCR, PER) or a combination of growth and amino acid deposition in muscle. It is not necessarily the optimal FM replacement level that maximizes WG and AA deposition in muscle. Therefore, we focused on the multi-objective optimization problem for the last two scenarios, growth and AA absorption in muscle. First, we determined the linear and quadratic regression equations for each parameter of the PP absorption of AA, growth of the fish, and deposition of AA in the fish muscles. Based on the R-square value, we identified that the quadratic regression was the best-fitted curve for all the parameters. This approach of curve fitting provided an overview regarding the interaction between FM replacement level and the AA absorption in blood or growth or AA deposition in muscle and helped us to identify where the pattern was aligned and where not. Then, we evaluated the performance of both species by classical optimization followed by two-phase fuzzy goal programming to determine the optimum replacement level for a synchronized utilization of AA.

## Materials and methods

### Experimental fish

Fingerlings of *L. rohita* (initial mean length 3.97±0.25 cm and initial mean weight 2.01±0.17 g ) and *M. vittatus* (initial mean length 3.87±0.22 cm and initial mean weight 3.22±0.41 g ) were bought from Naihati fish farm, North 24 Parganas, West Bengal, India. Ectoparasites were removed by the bath of the fingerlings in 3% NaCl solution for 15 min, followed by acclimatization for two weeks in experimental tanks. During acclimatization, the fingerlings were fed with a control diet (Table 2) twice daily at 5% of body weight.

### Formulation of feed

A blend of fish offal (FO), clotted slaughterhouse blood (SHB) (172 g), and cane molasses (148:172:62 g) were fermented by 20 mL of freshly collected whey as a microbial inoculant to prepare WFAPB to replace FM in the formulation of feed for the experimental fish. A detailed collection process of the ingredients, fermentation process, and proximate composition of WFAPB has been described earlier (Samaddar and Kaviraj, 2015). The proximate composition of the FM used in this study is given in Table 1. Details of the non-protein supplements used in the formulation of feed have been given in our previous work (Samaddar and Kaviraj, 2015; Samaddar et al., 2021).

The WFAPB was air-dried and used as a protein supplement to replace FM. Five experimental feeds were prepared by replacing dietary FM at 0% (T1, FM-based control feed) 25% (T2), 50% (T3), 75% (T4), and 100% (T5) with the WFAPB. The ingredients were adjusted with non-protein supplements, vitamins, and minerals to make all five experimental feeds as isoproteic, isolipidic, and isoenergetic (Table 2).

**Table 2:** Ingredients composition and proximate composition of feed

	$T_1$	$T_2$	$T_3$	$T_4$	$T_5$
FM replacement level (%)	0	25	50	75	100
Ingredients (%)					
Fishmeal	47.65	39.3	29.44	16.45	-
Fermented blend	-	13.5	29.44	50.45	77.05
Non protein supplements	48.35	43.2	37.12	29.1	18.95
vitamin and mineral mixtures	2	2	2	2	2
CMC and Cr2O3 (1:1)	2	2	2	2	2
Proximate composition (% dry matter basis)					
Crude protein	30.16	30.22	30.08	29.98	29.92
Crude fiber	8.37	8.55	9.16	9.49	10.08
Moisture	9.13	9.22	9.61	10.12	10.66
Ash	8.75	8.13	7.82	7.37	6.39
Nitrogen free extract	34.84	35.45	34.66	34.22	34.08
Lactic acid	1.76	3.07	4.11	6.57	9.67
Acetic acid	0.42	0.93	1.96	2.86	4.53
Gross energy (kJ g −1)	14.98	15.22	15.15	15.02	15
Free amino acid	0.07	0.15	0.31	0.41	0.52
Essential amino acids (EAA)					
Arg	2.13	2.07	1.87	1.73	1.53
His	1.52	1.43	1.39	1.22	1.03
Iso	2.03	1.88	1.66	1.51	1.23
Leu	2.62	2.85	3.06	3.28	3.63
Lys	2.37	2.38	2.48	2.6	2.76
Met	0.93	0.83	0.71	0.62	0.42
Phe	1.18	1.27	1.42	1.6	1.82
Thr	1.11	1.07	1.08	1.11	1.13
Val	1.51	1.65	1.69	1.68	1.71
Total EAA	15.37	15.41	15.34	15.33	15.29
Non essential amino acids					
Ala	1.44	1.47	1.76	20.2	2.23
Asp	1.82	2.12	2.33	2.63	2.59
Cys	0.22	0.23	0.23	0.26	0.29
Glu	2.68	2.64	2.77	2.14	2.97
Gly	1.45	1.56	1.32	1.28	1.15
Pro	1.03	1.02	1.06	1.06	0.99
Ser	0.72	0.67	0.69	0.64	0.58
Tyr	0.92	0.65	0.58	0.38	0.15
Total NAA	10.26	10.31	10.69	10.55	10.82

### Experimental design

Experiments were designed to evaluate feeding, apparent protein digestibility of the feed, postprandial AA absorption in plasma, growth, and AA deposition in the muscle of the experimental fish. The postprandial AA absorption in plasma was evaluated in 50 L glass aquaria in the laboratory, while growth experiments, including AA absorption in muscle, were evaluated in outdoor cement vats.

#### Laboratory experiments

Each laboratory glass aquarium (50 L) was filled with 45 L tube-well water supplied from an overhead tank and was stocked with five acclimatized fingerlings. Altogether, fifteen aquaria were used and set as per randomized block design so that there were three replicates for each treatment (Table 2). Each aquarium was stocked with 5 number of acclimatized fingerlings. The fish were fed a ration at 5% of their body weight per day. The daily ration was divided into equal installments, one given at 8:00 h and another at 16:00 h. Continuous aeration and daily water exchange at 50% were maintained during the trial. The mortality of fish was also checked on a daily basis. The experiments were continued for 15 days. Every day, the leftover feed samples were collected by siphoning, after 6 h of feeding, oven-dried ($60^{\circ }$C), weighed, and stored at −80^∘^C. The weight of the uneaten diets was subtracted from the total dry feed supply to obtain the actual feed intake rate (FIR). Fecal samples were collected by siphoning from each aquarium continuously at a 3–4 h interval for a period of 17 h after the removal of uneaten feeds. To minimize nutrient leaching, only fresh and intact feces were collected and dried to a constant weight at ${60^\circ }$C in an oven and weighed before storing at ${-80^\circ }$C, until they were used for determination of crude protein and Cr content to calculate apparent protein digestibility (APD) value. Apparent protein digestibility (APD) was calculated as follows: [100–100 $\times$ (% Cr in diet/% Cr in feces) $\times$ (% protein in feces/% protein in diet)]. After the 15-day laboratory experiments were completed, a few fish from each replicate were used to determine the level of essential and nonessential amino acid contents in plasma. For this purpose, fish in each aquarium were starved for 24 h, followed by feeding. After 6 h of feeding, the fish specimens were sampled from the aquaria for determination of plasma AA because postprandial AA was found to reach a peak between 4 and 8 h after feeding (Larsen et al., 2012; Samaddar and Kaviraj, 2015; Mente et al., 2021). The sampled fish were stunned by a blow to the head, and a blood sample was taken from the caudal vein using heparinized syringes The blood samples were centrifuged at 2000 g for 10 min at 4^∘^C. After that, the plasma was pooled and kept at $-80^{\circ }$C until it was further examined following the standard procedure as described in (Larsen et al., 2012; Samaddar et al., 2015).

#### Outdoor growth experiments

The growth experiments were done in 400 L cemented circular tanks (diameter 90 cm, average depth 45 cm), each containing 3.0 cm thick soil at the bottom, and 350 L, the same deep tube well used in the laboratory. Each tank was stocked with 20 numbers of acclimatized fingerlings. The fish were fed a ration of 5% of the body weight twice daily, once at 8:00 h and next at 16:00 h. Fish were bulk-weighed, and a ratio of 5% of the body weight of the fish was calculated for each tank. The quantity of the feed given was readjusted every 15 days after taking the bulk weight of the fish. About 50% of the water was renewed every week during the trials. At the end of the 90 days of outdoor growth experiments, all fish were sampled from the experimental tanks. The body weight and length of the sampled fish were determined. Growth performances were evaluated by weight gain (WG), feed conversion ratio (FCR), specific growth rate (SGR), and protein efficiency ratio (PER) (Sharf and Khan, 2023). Five fish from each replicate tank were randomly collected, and the skin was carefully removed from the underlying musculature. Muscle samples were collected, washed in distilled water, and preserved quickly at −80^0^C for later determination of the biochemical parameters.

### Analytical methods

Proximate composition (crude protein, crude lipid, moisture, and ash) analyses of the FM, feed, and fish muscle were determined by AOAC methods (Helrich, 1990; Gao et al., 2023). Nitrogen-free extract (NFE) was calculated by taking the sum of values for crude protein, crude lipid, moisture, and ash and subtracting this from 100 (Maynard et al., 1979; Giromini et al., 2017). Gross energy was calculated based on the methodology of (Brafield, 1985; Dalsgaard et al., 2023). Determinations of the essential and nonessential amino acids in the feed, centrifuged blood plasma supernatant, and carcass of the fish were carried out in the HPLC system (Waters, USA) following the method of Kwanyuen and Burton (2010). During the indoor experimental trials, daily water temperature (^∘^) and water pH measurements were carried out by a digital thermometer and a direct reading digital pH meter (Cybertronics, Kolkata), respectively. The dissolved oxygen level was determined by standard procedures (Rice et al., 2012). In addition to these parameters, free carbon dioxide, total alkalinity, total hardness, orthophosphate, nitrite, and ammonia nitrogen of water were recorded on a weekly basis following the standard procedures.

### Data analyses

All data were subjected to a one-way analysis of variance (ANOVA) followed by the least significant difference (LSD) test between mean values of dietary treatments (Zar, 1999). Differences were considered significant when *P* < 0.05. We used linear and quadratic regression curves estimated between the FM replacement levels and AA in blood, growth parameters (WG, SGR, FCR, and PER), and AA deposition in muscle. The best-fitted curve was determined by comparing the R-square value. Correlation coefficients were also determined to verify whether a statistically significant linear relationship existed between FM replacement levels and the observed values of different parameters. Quadratic regression is the most common and straightforward way to explore a non-linear relationship between variables (Khieokhajonkhet et al., 2022). Then, the classical optimization technique was used to obtain the optimal FM replacement level by solving the first-order derivative equal to zero. Note that if the second-order derivative is negative, the solution of the equation represents the maximum level. Finally, we used a two-phase fuzzy approach to solve a multi-objective optimization problem and obtain a Pareto-optimal solution with respect to a set of variables (Wu et al., 2015; Ali et al., 2020). We refer to the Appendix for a detailed explanation of the methodology.

**Table 3:** Free amino acids ($\mu$g ml^-1^) in plasma collected 6 h postprandial of *L. rohita* and *M. vittatus* fingerlings fed the experimental feed

	*L. rohita*					*M. vittatus*				
	T1	T2	T3	T4	T5	T1	T2	T3	T4	T5
EAA										
Arg	1.77^a^	2.75^f^	4.11^g^	4.18^h^	4.16^a^	2.41^a^	3.17^b^	3.82^c^	4.01^d^	3.73^e^
His	1.41^a^	3.22^e^	3.95^f^	4.25^g^g	3.73^c^	2.06^a^	3.82^b^	4.19^c^	4.96^d^	4.76^e^
Iso	0.12^a^	0.34^c^	0.42^e^	0.46^f^	1.08^g^	0.68^a^	1.2^b^c	1.65^c^	1.65^c^	1.66^c^
Leu	0.04^a^	0.10^bc^	0.15^c^	0.33^d^	0.57^e^	1.87^a^	2.13^b^	2.69^c^	2.85^c^	3.18^d^
Lys	0.36^a^	0.56^c^	0.62^f^	0.84^d^	1.37^g^	0.06^a^	0.19^b^	0.53^c^	1.19^d^	1.88^e^
Met	0.19^a^	0.23^c^	0.23^cd^	0.24^db^	0.22^c^	0.07^a^	1.15^b^	1.23^b^	1.87^c^	1.73^d^
Phe	1.33^a^	2.32^f^	2.55^g^	2.74^d^	2.84^h^	2.09^a^	2.78^b^	3.2^c^	3.37^d^	3.68^e^
Thr	0.02^a^	0.03^b^	0.03^b^	0.04^c^	0.04^c^	0.01^a^	0.01^a^	0.02^b^	0.02^b^	0.02^b^
Val	0.08^a^	1.05^f^	1.56^d^	1.65^g^	1.83^h^	0.48^a^	0.89^b^	1.31^c^	2.47^d^	2.68^e^
$\sum$ EAA	5.32	10.6	13.62	14.73	15.84	9.73a	15.34b	18.64c	22.39d	23.32e
NAA										
Ala	0.01^a^	0.01^a^	0.01^a^	0.01^a^	0.02^b^	0.01^a^	0.01^a^	0.01^a^	0.02^a^	0.02^a^
Asp	2.65^a^	2.92^f^	3.13^a^	3.22^a^	3.76^a^	2.57^a^	2.69^a^	2.99^a^	3.38^a^	3.89^a^
Cys	0.13^a^	0.23^c^	0.26^d^	0.24^cd^	0.38^f^	1.02^a^	2.74^b^	3.1^c^	3.34^d^	3.96^e^
Glu	0.24^a^	0.86^c^	0.88^c^	0.92^d^	0.97^e^	0.03^a^	0.07^b^	0.18^c^	0.27^d^	0.38^e^
Gly	0.16^a^	0.22^b^	0.29^c^	0.34^d^	0.42^e^	0.01^a^	0.02^b^	0.03^c^	0.03^c^	0.02^b^
Pro	0.12^a^	0.13^ab^	0.17^ab^	0.35^c^	0.22^d^	0.29^a^	0.46^b^	0.99^c^	1.31^d^	1.32^d^
Ser	0.57^a^	1.64^e^	1.57^f^	1.5^g^	1.23^h^	0.56^a^	1.13^b^	1.38^c^	1.1^b^	0.86^d^
Tyr	0.16^a^	0.65^f^	0.87^g^	1.33^h^	1.25^e^	1.52^a^	2.21^b^	2.77^c^	2.57^d^	2.37^e^
$\sum$ NAA	4.04	6.66	7.18	7.91	8.25	6.01a	9.33b	11.45c	12.02d	12.82e

**Dissimilar superscripts in a row indicate significant difference (***P* **< 0.05)**

**Table 4:** Growth of *L. rohita* and *M. vittatus* fed the experimental feed (maen ± S.D.)

	*L. rohita*					*M. vittatus*				
	$T_1$	$T_2$	$T_3$	$T_4$	$T_5$	$T_1$	$T_2$	$T_3$	$T_4$	$T_5$
WG (%)	289.92	292.64	288.23	270.6	257.62	232.71	237.21	239.64	230.27	221.71
	± 8.53^a^	± 4.72^a^	±12.44^a^	±3.37^c^	±11.43^b^	±4.63^a^	±5.91^a^	±4.6^a^	±4.26^a^	±3.77^b^
SGR	0.65	0.65	0.64	0.62	0.61	0.57	0.58	0.58	0.57	0.56
	±0.01^a^	±0.00^a^	± 0.01^a^	±0.00^b^	±0.01^b^	± 0.005^ab^	±0.01^a^	±0.005^a^	±0.005^ab^	0.00^b^
FCR	1.35	1.35	1.36	1.38	1.4	1.42	1.4	1.38	1.43	1.48
	± 0.06^a^	±0.02^ab^	±0.07^ab^	±0.03^ab^	±0.09^b^	±0.03^ac^	±0.02^ac^	± 0.04^c^	± 0.01^ac^	±0.01^b^
PER	2.46	2.46	2.43	2.4	2.39	2.34	2.37	2.4	2.33	2.25
	±0.02^a^	± 0.01^a^	± 0.03^ab^	±0.01^ab^	±0.0^b^	± 0.01^ab^	±0.01^ab^	± 0.01^b^	± 0.01^a^	± 0.005^c^

**Dissimilar superscripts in a row indicate significant difference (***P* **< 0.05)**

## Results

The average ranges of temperature, pH, and dissolved oxygen recorded in water during the laboratory experiments were 28–30^0^C, 7.01–7.08, 5.12–6.46 mg / L, respectively. There was no mortality during the experiment. Mean feed intake rate (FIR) in *L. rohita* and * M. vittatus* in treatments T1, T2, T3, T4, T5 were 4.34, 4.41, 4.38, 4.16, 3.98 and 3.71, 3.71, 3.69, 3.68,3.66 g.100g^-1^ body wt. Day^-1^, respectively. Apparent protein digestibility (APD%) in T1, T2, T3, T4, and T5 was 81.80, 82.31, 82.47, 82.78, 83.05 in *L. rohita* and 84.77, 84.99, 85.44, 85.85, 86.24 in *M. vittatus*, respectively. FIR of fish fed WFAPB supplemented feed (T2–T5) did not vary significantly from the FM-based control feed (T1) in *L. rohita* up to 50 % FM replacement level (T3) and in * M. vittatus* up to 75% replacement level (T4). In *L. rohita* FIR significantly reduced at T4 and T5 and in *M. vittatus* the FIR significantly reduced only at T5. APD (%) in both fishes significantly increased from 50% replacement level (T3) onward. Dietary effects on postprandial concentration of FAA in plasma (6 h after meal) have been presented in Table 3.

Uptake of free EAA and NAA in plasma significantly increased in WFAPB supplemented feed (T2–T5) as compared to FM-based feed (T1) in both *L. rohita* and *M. vittatus*. The uptake rate of free AA in plasma was higher in *M. vittatus* than in *L. rohita*. The uptake pattern of both EAA and NAA varied with individual AA and differed between the two species.

**Table 5:** Proximate composition of carcass (% dry weight) of *L. rohita* and *M. vittatus* fed the experimental feed

	*L. rohita*						*M. vittatus*					
	Initial	T1	T2	T3	T4	T5	Initial	T1	T2	T3	T4	T5
Crude Protein	9.98^a^	11.64^b^	11.65^b^	11.59^b^	11.3^c^	11.2^c^	14.15^d^	16.29^e^	16.35^ef^	16.38^f^	16.25^eg^	16.14^g^
Crude Lipid	2.75^a^	3.56^b^	3.62^b^	3.67^b^	3.57^b^	3.32^b^	4.37^c^	4.71^d^	4.95^d^	4.1^e^	4.16^de^	4.05^d^
Ash	1.84^a^	3.28^b^	3.29^b^	3.24^b^	3.17^c^	3.08^d^	2.66^e^	3.93^f^	3.95^f^	3.91^f^	3.89^fg^	3.86^g^

**Dissimilar superscripts in a row indicate significant difference (***P* **< 0.05)**

**Table 6:** Amino acid (AA) deposition (% dry matter basis) in the muscle of *L. rohita* and *M. vittatus* fed WFAPB supplemented feed for 90 days

	*L. rohita*						*M. vittatus*					
EAA	Initial	$T_1$	$T_2$	$T_3$	$T_4$	$T_5$		$T_1$	$T_2$	$T_3$	$T_4$	$T_5$
Arg	3.98^a^	4.41^b^	4.44^bc^	4.4^b^	4.32^d^	4.1^f^	3.92^a^	4.41^b^	4.48^c^	4.7^d^	4.41^b^	4.25^e^
Hist	2.04^a^	2.27^b^	2.3^b^	2.23^bc^	2.21^c^	2.13^d^	1.32^a^	1.49^b^	1.5^b^	1.59^c^	1.45^bd^	1.42^e^
Iso	2.05^a^	2.62^b^	2.65^c^	2.6^b^	2.54^d^	2.41^e^	2.49^a^	2.85^bd^	2.88^b^	2.95^c^	2.87^b^	2.73^f^
Leu	4.44^a^	4.93^b^	4.97^c^	4.91^b^	4.81^d^	4.68^e^	2.24^a^	2.57^bd^	2.55^bd^	2.7^c^	2.56^b^	2.41^e^
Lys	4.86^a^	5.43^b^	5.44^bc^	5.42^b^	5.25^f^	5.12^g^	6.04^a^	6.87^b^	6.89^b^	7.18^c^	6.84^b^	6.55^d^
Met	0.84^a^	1.01^b^	1.07^e^	0.97^f^	0.98b^f^	0.93^d^	1.74^a^	2.03^b^	2.0^d^	2.13^f^	2.01^d^	1.87^e^
Phe	4.37^a^	4.88^b^	4.9^b^	4.8^b^	4.71^f^	4.57^g^	2.61^a^	2.95^b^	2.96^b^	3.2^c^	2.91^d^	2.83^e^
Thr	2.57^a^	2.87^b^	2.91^f^	2.86^b^	2.7^d^	2.7^d^	2.58^a^	2.92^b^	2.99^c^	3.13^d^	2.9^d^	2.82^e^
Val	0.66^a^	0.73^b^	0.76^bc^	0.71^bd^	0.7^d^	0.65^e^	2.85^a^	3.27^b^	3.29^b^	3.47^f^	3.23^d^	3.04^e^
$\sum$ EAA	26.25	29.12	29.45	28.92	28.18	27.28	25.81	29.26	29.41	30.98	29.21	27.85
Ala	3.82^a^	4.74^b^	4.76^b^	4.71^f^	4.13^d^	4.03^e^	3.83^a^	4.37^b^	4.33^e^	4.77^c^	4.37^b^	4.17^d^
Asp	5.8^a^	6.49^b^	6.55^c^	6.45^f^	6.3^d^	6.1^g^	5.93^a^	6.66^b^	6.61^b^	7.08^c^	6.6^b^	6.37^d^
Cys	0.16^a^	0.29^b^	0.31^b^	0.26^d^	0.22^e^	0.21^e^	0.51^a^	0.56^b^	0.57^cb^	0.59^c^	0.56^b^	0.49^d^
Glu	6.06^a^	6.78^b^	6.8^c^	6.73^f^	6.61^d^	6.41^e^	8.74^a^	10.05^b^	10.01^be^	10.45^c^	9.98^e^	9.48^d^
Gly	5.45^a^	6.09^bf^	6.11^f^	6.06^b^	5.9^d^	5.76^e^	4.9^a^	5.62^b^	5.66^b^	5.98^c^	5.54^d^	5.39^e^
Pro	2.94^a^	3.28^b^	3.29^bc^	3.19^eg^	3.17^g^	3.08^f^	2.96^a^	3.41^bc^	3.42^bc^	3.47^d^	3.41^bc^	3.21^e^
Ser	2.03^a^	3.16^b^	3.18^bc^	3.11^e^	3.02^d^	2.96^d^	2.95^a^	3.33^b^	3.32^b^	3.52^c^	3.25^d^	3.15^e^
Tyr	1.37^a^	1.53^bf^	1.55^f^	1.52^b^	1.42^e^	1.41^e^	1.99^a^	2.24^b^	2.27^b^	2.37^c^	2.27^b^	2.18^e^
$\sum$ NAA	28.05	32.39	32.37	32.03	30.74	30.03	29.87	36.24	36.4	38.17	36.07	34.46

**Dissimilar superscripts in a row indicate significant difference (***P* **< 0.05)**

Water quality parameters (temperature, pH, dissolved oxygen, free carbon dioxide, total alkalinity, total hardness, orthophosphate, nitrite, and ammonia nitrogen) observed during the 90-day experiment were within the acceptable limits. Table 4 presents the growth of *L. rohita* and *M. vittatus* fed the WFAPB-supplemented feed for 90 days. WG increased only up to 25% replacement level in *L. rohita* and up to 50% replacement level in *M. vittatus*. SGR did not vary significantly from control (T1) up to 50% replacement level (T3) in *L. rohita* and decreased thereafter, while it did not vary significantly from control (T1) up to 75% replacement level (T3) in *M. vittatus* and decreased thereafter. In both the species, FCR and PER remained unchanged up to 75% replacement level (T4) and decreased at 100% replacement level (T5).

**Table 7:** Regression equations for postprandial concentration of free amino acids ($\mu$g ml^-1^) in plasma 6 h after feeding of WFAPB supplemented feed by *L. rohita* and *M. vittatus*

	*L. rohita*				*M. vittatus*			
EAA	Equation	R^2^	$\frac{d^2X}{dS^2}$	Dose	Equation	R^2^	$\frac{d^2X}{dS^2}$	Dose
Arg	2.152+0.024840*S*	0.81			2.732+0.01392*S*	0.72		
	1.682+0.062440*S* $-$0.000376$S^2$	0.97	$-$0.0008	83.03	2.369+.04295*S* $-$0.000290$S^2$	0.99	$-$0.0006	73.98
Hist	2.178+0.022680*S*	0.63			2.650+0.02616*S*	0.80		
	1.451+0.080851*S* $-$0.000582$S^2$	1.00	$-$0.0012	69.50	2.147+0.06639*S* $-$0.000402$S^2$	0.97	$-$0.0008	82.51
Iso	0.076+0.008160*S*	0.81			0.886+0.00964*S*	0.78		
	0.185$-$0.000526*S*+0.000087$S^2$	0.89	0.0002	3.03**(100)**	0.676+0.02644*S* $-$0.000168$S^2$	0.99	$-$0.0003	78.69
Leu	$-$0.020+0.005160*S*	0.90			1.876+0.01336*S*	0.97		
	0.050$-$0.000440*S*+0.000056$S^2$	0.99	0.0001	3.93**(100)**	1.839+0.01633*S* $-$0.000030$S^2$	0.98	$-$0.0001	274.81**(100)**
Lys	0.290+0.009200*S*	0.89			$-$0.158+0.01856*S*	0.93		
	0.407$-$0.000171*S*+0.000094$S^2$	0.97	0.0002	0.91**(100)**	0.048+0.00210$-$0.000165$S^2$	1.00	0.0003	6.39
Met	0.208+0.000280*S*	0.33			0.402+ 0.01616*S*	0.81		
	0.192+0.001537*S* $-$0.000013$S^2$	0.92	$-$0.00002	60.99	0.133+0.03765*S* $-$0.000215$S^2$	0.94	$-$0.0004	87.61
Phe	1.668+0.013760*S*	0.80			2.270+0.01508*S*	0.94		
	1.408+0.034560*S* $-$0.000208$S^2$	0.96	$-$0.0004	83.08	2.126+0.02662*S* $-$0.000115$S^2$	0.99	$-$0.0002	115.32**(100)**
Vel	0.414+0.016400*S*	0.84			0.370+0.02392*S*	0.95		
	0.128+0.039257*S* $-$0.000229$S^2$	0.98	$-$0.0005	85.86	0.419+0.02003*S*+0.000039$S^2$	0.95	0.0001	$-$257.80**(100)**
Asp	2.632+0.010080*S*	0.93			2.438+0.01332*S*	0.95		
	2.692+0.005280*S*+0.000048$S^2$	0.95	0.0001	$-$55.00 **(100)**	2.562+0.00338*S*+0.000099$S^2$	1.00	0.0002	$-$16.98**(100)**
Cys	0.146+0.002040*S*	0.82			1.536 0.02592	0.86		
	0.150+0.001697*S*+0.000003$S^2$	0.82	0.0001	$-$249.56(**(100)**)	1.205+0.05243*S* $-$0.000265$S^2$	0.94	$-$0.0005	98.88
Glu	0.470+0.006080*S*	0.64			0.006+0.00360*S*	0.98		
	0.310+0.018880*S* $-$0.000128$S^2$	0.88	$-$0.0003	73.75	0.023+0.00223*S*+0.000014$S^2$	0.99	0.00002	$-$81.25**(100)**
Gly	0.158+0.002560*S*	0.68			0.016+0.00012*S*	0.32		
	0.161+0.002331*S*+0.000002$S^2$	0.89	0.000004	$-$506.74**(100)**	0.009+0.00069*S* $-$0.000006$S^2$	0.96	$-$0.00001	60.50
Pro	0.114+0.002*S*	0.503			0.292+0.012*S*	0.926		
	0.292+0.012*S* $-$0.000016 $S^2$	0.543	$-$0.000003	$-$506.74**(100)**	0.216+0.018*S* $-$0.000061$S^2$	0.947	$-$0.00001	60.50
Ser	1.066+0.004720*S*	0.18			0.892+0.00228*S*	0.08		
	0.683+0.035349*S* $-$0.000306$S^2$	0.85	$-$0.0006	57.70	0.585+0.02685*S* $-$0.000246$S^2$	0.94	$-$0.0005	54.64
Try	0.280+0.011440*S*	0.90			1.876+0.00824*S*	0.46		
	0.151+0.021726*S* $-$0.000103$S^2$	0.97	$-$0.0002	105.57**(100)**	1.513+0.03727*S* $-$0.00029$S^2$	0.97	$-$0.0006	64.19

Note: $S\in (0,100)$ represent the FM replacement level. For minimization parameters, after the calculated dose, the absorption will increase with the increasing FM replacement level. For instance, *Iso* is minimum at 3.03%. Therefore, the maximum FM replacement that maximizes the absorption of *Iso* is at 100%

Bold number represent the highest or least value other than maximum or minimum

The biochemical composition of fish muscle before and after the experimental trials has been presented in Table 5.

Levels of crude protein (CP), crude lipid (CL), and ash contents (AC) increased significantly in all treatments in comparison to initial values. *L. rohita* reared for 90 days did not show any change in CP level and ash content up to 50% FM replacement level (T3) but decreased significantly with the increase of dietary FM replacement level (T4 and T5). CL level of fish muscle remained unchanged in all groups. In *M. vittatus*, CP and CL values were significantly high in T3 (50% replacement level) and remained unchanged up to T4 (for CP) and T5 (for CL), respectively, in comparison to control, while AC decreased significantly only in T5.

Deposition of essential and non-essential amino acids (EAA and NAA) after 90 days of feeding of the WFAPB supplemented feed has been presented in Table 6.

The quantity of EAA and NAA significantly increased in fish muscle from the initial value after 90 days of rearing. Total EAA content was initially significantly higher in *L. rohita* but after 90 days experimental trial *M. vittatus* showed comparable status of total EAA with *L. rohita* up to 25% replacement level (T2) and the total EEA increased thereafter. Total NAA content in the muscle of *M. vittatus* was significantly higher in all treatments as compared to *L. rohita*. Deposition of all EAA and NAA in body muscle significantly reduced at T4 (75% replacement level) in *L. rohita* and at T5 (100% replacement level) in *M. vittatus*. indicating that *M. vittatus* was more efficient than *L. rohita* to utilize the EAAs and NAAs. *M. vittatus* showed maximum deposition of EAAs and NAAs in T3 (50% replacement level), while *L. rohita* showed maximum deposition of EAA and NAA in T2 (25% replacement level) with isoleucine, leucine, methionine, threonine (EAA), and glutamic acid (NAA) exhibiting significantly higher deposition at T2 than T1, the rest showing comparable values between T1 and T2. Except for histidine and leucine among EAA and alanine among NAA, deposition of all AAs was significantly higher in *M. vittatus* as compared to *L. rohita*. We determined linear and quadratic regression curves for postprandial concentration of free AA in plasma, growth, and AA deposition in muscle. We determined the optimal FM replacement level for each variable.

**Fig. 1:**
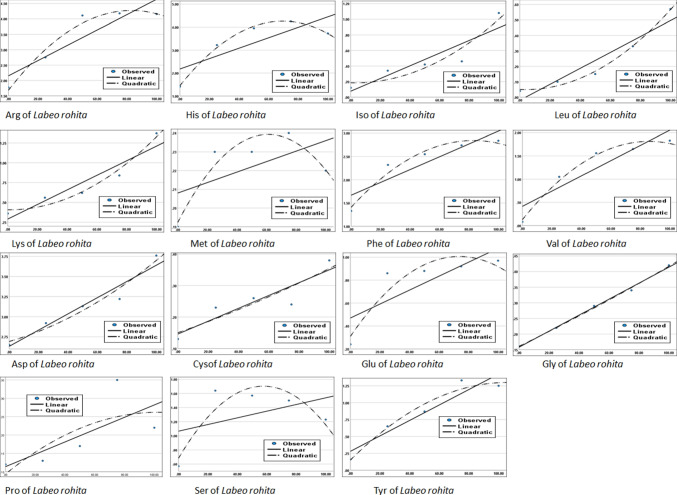
Linear and quadratic regressions of PP absorption of AA in plasma of L. rohita

**Fig. 2:**
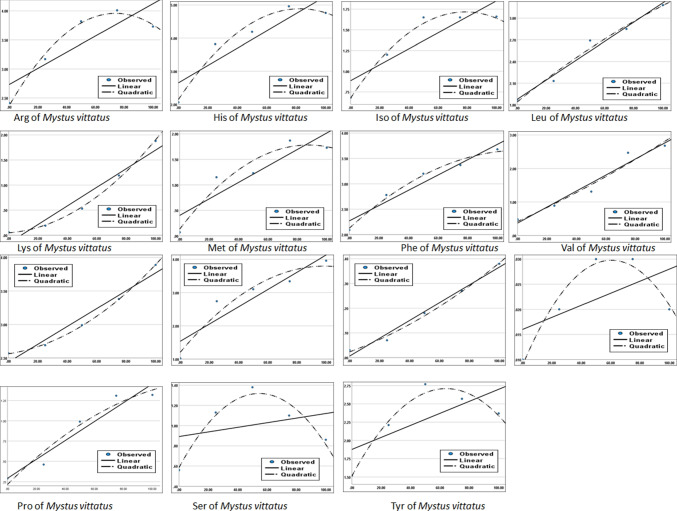
Linear and quadratic regressions of PP absorption of AA in plasma of M. vittatus

**Fig. 3:**
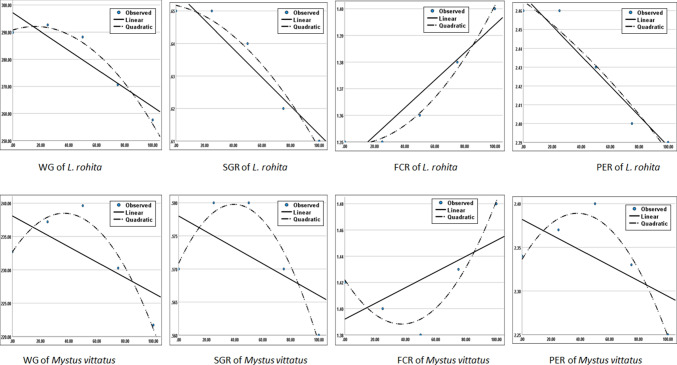
Linear and quadratic regressions of four growth parameters of *L. rohita* and *M. vittatus*

From R-square values, we see that the quadratic curves are best fitted for postprandial absorption of free AAs in plasma (see Table 7 and Figs. 1 and 2 in the Appendix). Quadratic regression followed by classical optimization indicates that optimal FM replacement level for postprandial free AA uptake in plasma ranges from 57.70 to 100% and 60.05 to 100%, for *L. rohita* and *M. vittatus*. But when we applied the same technique for the four growth parameters (see Table 11 and Fig. 3 in the Appendix), the corresponding optimal FM replacement label changed.

**Fig. 4:**
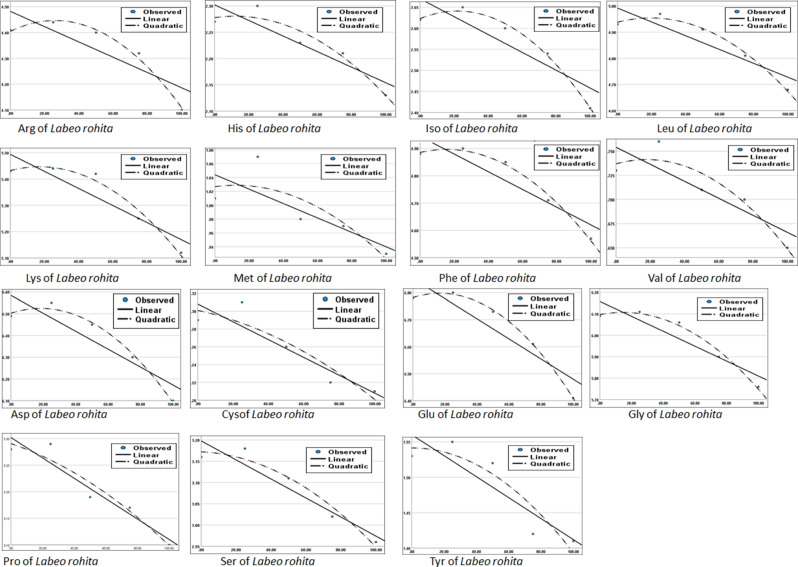
Linear and quadratic regressions of AA deposition in muscle of *L. rohita*

**Fig. 5:**
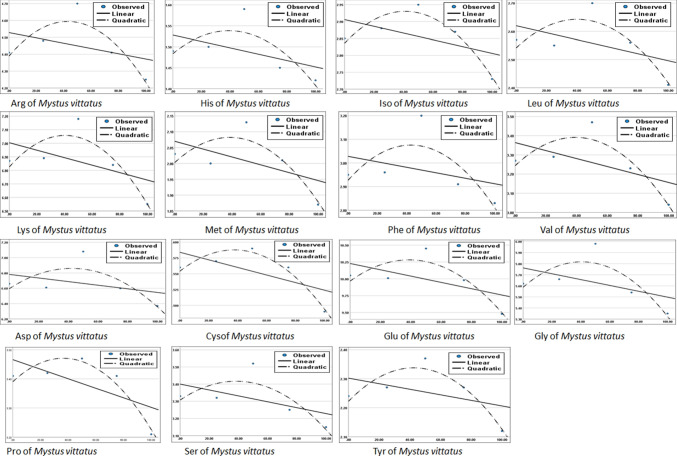
Linear and quadratic regressions of AA deposition in muscle of *M. vittatus*

The optimal FM levels are not unique; the WG is maximum at 16.02%, whereas FCR is minimum at 4% for *L. rohita*. Noticeably, the optimal FM levels for SGR and PER is $-$14.27% and $-$124.22%, respectively. From Fig. 3 in the Appendix, we also observe that both SGR and PER decrease with the increase of FM replacement level, indicating adverse effects of FM replacement by WFAPB. In summary, it is found that the optimum FM replacement level for different parameters ranges from 0 to 16.02% in *L. rohita* while that for *M. vittatus* was quite narrow and ranges from 36.64 to 37.58%. Therefore, based on growth parameters *M. vittatus* appears to utilize WFAPB supplemented feed better than *L. rohita*. Next, we determined the optimal replacement level based on the deposition of EAA and NAA in fish muscle after 90 days of rearing (see Table 9 and Figs. 4 and 5 in the Appendix).

The optimal deposition of all AAs in the muscle was found in the replacement levels 0 to 26.02% for *L.rohita*. Ser and Cys continuously decreased with increasing FM replacement level, and 0% (FM-based control feed) was the best fit. Therefore, if we compare the optimum FM replacement level required for growth and for AA deposition in muscle, it is revealed that these are quite different, though *M. vittatus* exhibiting a close optimal replacement level for AA deposition (37.5–42.96%) with that of growth (36.4 to 37.58%).

The correlation coefficient between FM replacement levels and all variables (postprandial absorption of AA, growth, and AA deposition in muscle) are presented in Tables 10, 11, and 12. FM replacement levels showed significant negative correlations with WG, SGR, and PER and strong significant positive correlations with FCR of *L. rohita*. *M. vittatus* showed a similar trend of correlations between FM replacement levels and the growth parameters, but the correlations were insignificant (see Tables 11 in the Appendix).

## Discussion

Stimulation of protein synthesis is the key factor behind the ideal growth of fish (Li et al., 2009). All EAA and NAA are required in the right proportion during protein synthesis. Deficiency of a particular AA may result in the metabolism of other AA for energy, leading to poor retention of protein in the body of fish (Hardy, 2010; Ambardekar et al., 2009; Brezas and Hardy, 2020; Mente et al., 2021). Fish growth depends on the availability of EAA and NAA, the pattern of their uptake in blood, and deposition in muscle as protein. After absorption in the blood, AA are transported to the liver for protein synthesis and subsequent transformation into muscle resulting in growth. While the crude protein level of muscle serves as an indicator of growth, the level of EAA and NAA in the muscle indicates the efficiency of the fish in utilizing AA from the feed.

We aimed to evaluate if the replacement of FM by WFAPB could serve as a balanced source of AA that can be easily absorbed and utilized in the body of fish. The notion behind this was to reduce the cost and dependency on FM as a protein source and use of fermented animal protein blend as an ideal FM replacer (Samaddar et al., 2021; Bai et al., 2021; Achmad et al., 2023), and use of whey as an easily available inoculum to ferment the blend. Therefore, we determined the efficiency of the experimental feed at different FM replacement levels by three performances of fish: (i) postprandial (6 h) uptake of free amino acids, (ii) growth of the fish after 90 days of rearing, and (iii) quantitative and qualitative deposition of the essential and non-essential AA in the muscle after 90 days of rearing. We used quadratic regression followed by classical optimization to evaluate the performances to determine the optimal FM replacement level. For postprandial uptake of free AA, we did not make any regression for tryptophan (EAA) and alanine (NAA) because of their poor absorption in plasma.

Maximum or minimum absorption of the AA in plasma has been presented in Table 7 and Figs. 1 and 2 in the Appendix. In *L. rohita*, maximum absorption of Arg, Hist, Met, Phe, Val, Glu, and Ser occurred at replacement level 50 to 80% and decreased after that, while maximum absorption of tyrosin was obtained at 100% replacement level. In *M. vittatus*, maximum absorption of Gly, Ser, and Tyro was obtained between 50 and 60% replacement level. In contrast, Arg, Hist, Iso, and Met absorption peaked at $\ge$ 80% replacement level and Leu, Phe, and Cys peaked at 100% replacement level. Results revealed that 60 to 80% replacement of FM by WFAPB could maximize five EAAs and three NAAs in *L. rohita* and six EAAs and four NAAs in *M. vittatus*. Even 100% replacement was possible for tyrosin in *L. rohita* and for leucin, phenylealanine and cysteine in *M. vittatus*. It is established that the free concentration of an individual AA in blood will remain low until its requirements are met, and therefore, changes in the postprandial level of free amino acid represent an important criterion to determine amino acid requirements of animals (Mente et al., 2021). This study indicates hyperaminoacidemic conditions of some AA in the blood of fish fed the WFAPB-supplemented feed. Numerous efforts have been made towards the determination of postprandial concentrations of FAA in different fish species fed diverse dietary ingredients (Mente et al., 2003; Larsen et al., 2012; McCarthy and Fuiman, 2011; Xu et al., 2016; Mente et al., 2021). Juvenile rainbow trout (*Oncorhynchus mykiss*) fed a more growth-supportive FM diet exhibited a synchronized pattern of plasma AA while it appeared less synchronized in those fed plant meal diets (Larsen et al., 2012). When AA is not available in required quantities and in a synchronized pattern, cellular protein synthesis leads to alternative metabolization for energy (Brezas and Hardy, 2020).

**Table 8:** Regression equations for four growth parameters of *L. rohita* and *M. vittatus* fed WFAPB supplemented blend for 90 days

	*L. rohita*				*M. vittatus*			
EAA	Equation	R^2^	$\frac{d^2X}{dS^2}$	Dose	Equation	R^2^	$\frac{d^2X}{dS^2}$	Dose
WG	297.13 $-$0.34656*S*	0.82			238.096 $-$0.11576*S*	0.43		
	290.7557+ 0.16338 *S* $-$0.0050994$S^2$	0.98	$-$0.010198	16.02	232.6788571+ 0.31761*S* $-$0.004334$S^2$	0.96	$-$0.008668	36.64
SGR	0.656 $-$0.00044*S*	0.92			0.578 $-$0.00012*S*	0.32		
	0.651714286 $-$0.00010*S* $-$0.0000034$S^2$	0.97	$-$0.000007	$-$14.28**(0)**	0.570857143+ 0.00045*S* $-$0.000006$S^2$	0.96	$-$0.000012	37.58
FCR	1.342+0.00052*S*	0.90			1.392+ 0.00060	0.40		
	1.349142857 $-$0.00005*S* + 0.0000057$S^2$	0.99	0.000011	4.51	1.422$-$0.00180*S*+0.000024$S^2$	0.95	0.000048	37.50
PER	2.468 $-$0.00080*S*	0.93			2.382 $-$0.00088*S*	0.38		
	2.465142857 $-$0.00057*S* $-$0.0000023$S^2$	0.94	$-$0.000005	$-$124.22**(0)**	2.336285714+0.00278*S* $-$0.000037$S^2$	0.96	$-$0.000074	37.53

Note: $S\in (0,100)$ represent the FM replacement level. For maximization parameters, after the calculated dose, the absorption will decrease with the increased FM replacement level. For instance, *SGR* is maximum at $-$14.28%. Therefore, the maximum FM replacement that maximize *SGR* is at 0%

Bold number represent the highest or least value other than maximum or minimum

To date, we do not have any report on the amino acid profile of the feed with FM replaced by WFAPB, nor do we have any report on the AA absorption pattern in blood and its subsequent deposition in muscle. This study reveals that the higher the level of FM replacement by WFAPB, the more the availability of FAAs in feed because fermentation leads to the release of excess FAA (Espe and Lied, 1994; Bai et al., 2021). Results of the present study indicate that 60 to 80% replacement could be done to maximize absorption of most of the EAA and NAA in plasma, but it was unsuitable for optimum growth of the fish.

**Table 9:** Amino acid (AA) deposition (% dry matter basis) in the muscle of *L. rohita* and *M. vittatus* fed WFAPB supplemented blend for 90 days

	*L. rohita*				*M. vittatus*			
AA	Equation	R^2^	$\frac{d^2X}{dS^2}$	Dose	Equation	R^2^	$\frac{d^2X}{dS^2}$	Dose
Arg	4.48$-$0.002960*S*	0.72			4.53 $-$0.00156	0.14		
	4.40+0.003211*S* $-$0.00006$S^2$	0.99	$-$0.00012	26.02	4.39+0.00953*S* $-$0.00011$S^2$	0.77	$-$0.00022	42.9666
Hist	2.30$-$0.001480*S*	0.81			1.53$-$0.00076*S*	0.22		
	2.28+0.000463*S* $-$0.00002$S^2$	0.93	$-$0.00004	11.93	1.48+0.00278*S* $-$0.00004$S^2$	0.63	$-$0.00007	39.2655
Iso	2.67$-$0.002120*S*	0.78			2.91$-$0.00100*S*	0.24		
	2.62+0.001651*S* $-$0.00004$S^2$	0.99	$-$0.00008	21.90	2.84+0.00460*S* $-$0.00006*S*	0.92	$-$0.00011	41.0714
Leu	4.99$-$0.002640*S*	0.80			2.62$-$0.00124*S*	0.23		
	4.94+0.001703*S* $-$0.00004$S^2$	0.99	$-$0.00009	19.62	2.54+0.00505*S* $-$0.00006$S^2$	0.74	$-$0.00013	40.1431
Lys	5.49$-$0.003240*S*	0.81			7.00$-$0.00276*S*	0.24		
	5.43+0.001674*S* $-$0.00005$S^2$	0.98	$-$0.00010	17.05	6.83+0.01153*S* $-$0.00014$S^2$	0.80	$-$0.00029	40.3429
Met	1.04$-$0.001040*S*	0.62			2.07$-$0.00124*S*	0.28		
	1.03 0.000331*S* $-$0.00001$S^2$	0.72	$-$0.00003	12.04	2.00+0.00413*S* $-$0.00005$S^2$	0.74	$-$0.00011	38.4544
Phe	4.94$-$0.003240*S*	0.84			3.03$-$0.00116*S*	0.11		
	4.89+0.001446*S* $-$0.00005$S^2$	0.99	$-$0.00009	15.42	2.93+0.00695*S* $-$0.00008$S^2$	0.58	$-$0.00016	42.8483
Vel	0.75$-$0.000880*S*	0.73			3.36$-$0.00208*S*	0.29		
	0.74+0.000491*S* $-$0.00001$S^2$	0.89	$-$0.00003	17.92	3.24+0.00752*S* $-$0.00010$S^2$	0.82	$-$0.00019	39.1667
Asp	6.58$-$0.004120*S*	0.81			6.78$-$0.00236*S*	0.13		
	6.50+ 0.002394*S* $-$0.00007$S^2$	0.99	$-$0.00013	18.39	6.59+0.01261*S* $-$0.00015$S^2$	0.59	$-$0.00030	42.1176
Cys	0.31$-$0.001000*S*	0.84			0.58$-$0.00060*S*	0.39		
	0.30$-$0.000429*S* $-$0.00001$S^2$	0.86	$-$0.00001	$-$37.63**(0)**	0.55+0.00180*S* $-$0.00002$S^2$	0.94	$-$0.00005	37.5
Glu	6.85$-$0.003720*S*	0.83			10.23$-$0.00468*S*	0.29		
	6.78+ 0.001880*S* $-$0.00006$S^2$	1.00	$-$0.00011	16.79	9.97+0.01623*S* $-$0.00021$S^2$	0.79	$-$0.00042	38.8092
Gl	6.16$-$0.003480*S*	0.84			5.76$-$0.00264*S*	0.21		
	6.10+0.001434*S* $-$0.00005$S^2$	0.99	$-$0.00010	14.60	5.59+0.01130*S* $-$0.00014$S^2$	0.71	$-$0.00028	40.5308
Pro	3.302$-$0.002*S*	0.91			3.466$-$0.002*S*	0.41		
	3.291–0.001*S* $-$0.000009 $S^2$	0.93	$-$0.000018	14.60	3.390+0.004*S* $-$0.00006$S^2$	0.92	$-$0.00012	40.5308
Ser	3.20$-$0.002240*S*	0.89			3.40 $-$0.00172*S*	0.25		
	3.17 $-$0.000183*S* $-$0.00002$S^2$	0.96	$-$0.00004	$-$4.44(0)	3.31+0.00571*S* $-$0.00007$S^2$	0.66	$-$0.00015	38.4253
Try	1.56$-$0.001480*S*	0.79			2.30$-$0.00096*S*	0.18		
	1.54+0.000006*S* $-$0.00001$S^2$	0.86	$-$0.00003	0.20	2.22 0.00544*S* $-$0.00006$S^2$	0.88	$-$0.00013	42.5

Bold number represent the highest or least value other than maximum or minimum

In fact, quadratic regression followed by classical optimization reveals higher WG of *L. rohita* and *M. vittatus* only at 16.02% and 36.64% replacement level, respectively (Table 8). Applying the same technique on the deposition of AA in muscle, it is revealed that a maximum 26.02% replacement of FM by WFAPB in feed is possible for *L. rohita* and 42.96% replacement is possible for *M. vittatus* (Table 9). *M. vittatus* having a functional acid-peptic stomach could digest and utilize more protein than the stomach-less fish *L.rohita* and thus a higher level of FM replacement by WFAPB was possible for *M. vittatus*. However, comparing the optimal FM replacement level for the three performances of the fish, the postprandial uptake of AA, growth, and AA deposition in muscle, it is revealed that the range of optimal replacement varies widely from one performance to another. However, it is clear that 60–80% FM replacement level results in hyperaminoacidic condition in the blood of both fish and results in the reduction of growth.

Therefore, we attempted to determine the optimal FM replacement level that maximizes the growth and deposition of AA in muscle. In practice, the growth and AA deposition in muscle are crucial parameters to determine the production and quality of the fish muscle. Fish growth depends on the quantity and quality of the AA available and how they are utilized in the synthesis of protein. The optimization approach we proposed provides a balanced amount of all essential amino acids for the optimum growth of fish. Combing the ranges of optimal FM replacement level for both growth and AA deposition in muscle, we observed that within 0$-$26.02%, either one of the growth parameters (WG, SGR, FCR, PER) or AA deposition was optimal for *L. rohita*, whereas the range is 36.64%$-$42.96% for *M. vittatus* indicating higher efficiency of *M.viitatus* to utilize the whey fermented blend ingredients than *L. rohita*. Noticeably, Zhu et al. (2021) also reported that different growth parameters of shrimp respond differently to different levels of FM replacement.

Now the question is: what is the optimal replacement if all the parameters are taken into consideration? For this purpose, we used two-phase fuzzy goal programming. We refer to Solution procedure for two-phase fuzzy goal programming section in the Appendix for the detailed implementation of the methodology. The main advantage of the two-phase approach is that the methods can ensure not only the Pareto optimal solution but also a compromise solution. We were applying this procedure on data of growth and AA deposition in the muscle of *L. rohita*, we obtained the replacement label 7.63% and 12.23%, respectively, for growth parameters (all four growth parameters) and all parameters taken together (four growth parameters and fifteen AA deposition parameters). Similarly, for *M. vittatus* we obtained the replacement label 36.79% and 40.02%, respectively, for only growth parameters and all parameters taken together. It strongly establishes that *M. vittatus*, being a carnivorous stomach-bearing fish, is superior to *L. rohita* in utilizing WFAPB-supplemented feed. This was also established previously when these two species of fish were reared on feed supplemented by *Lactobacillus acidophilus* fermented animal protein blend (Samaddar and Kaviraj, 2015; Samaddar et al., 2021). *M. vittatus* was more efficient in using the fermented blend than *L. rohita*. Carnivorous fish can efficiently utilize animal protein blends. Langi et al. (2023) observed that the pike perch *Sander lucioperca*, a strong carnivorous fish, could successfully utilize 40% replacement of FM by a poultry-based protein combining feather meal, poultry meat, and bone meal. The present study further establishes that *M. vittatus* can better utilize the feed supplemented by whey fermented animal protein blend than the *Lactobacillus acidophilus* fermented animal protein blend. 36 to 41% FM replacement by WFAPB will also not likely to cause hyperaminoacidic conditions in plasma.

In several urban and rural areas of India, two of the commonly generated animal by-products are FO and SHB (Wangkheirakpam et al., 2019), which have been considered as effective dietary fishmeal replacer in the formulation of feed for several fish species after processing through microbial fermentation (Samaddar et al., 2021; Siddik et al., 2020). However, considering limited knowledge, facilities, infrastructure, and availability of resources, especially in rural areas, an easy fermentation tool could be a sustainable solution for poor and marginal fish farmers to produce fish feed with available local resources. Besides substrate in a fermentation media, a suitable and effective inoculum serves as the key component. Whey is a pollution-causing by-product generated from dairy industries across the Indian subcontinent (Zandona et al., 2021; Das et al., 2016). Recycling of this by-product as fermentation inoculum has been considered to be a successful bioconversion technique for improving nutritional properties and shelf life of FO and SHB (Chowdhury et al., 2022). Such characteristics make it a potential and easily accessible source of microorganisms for rural farmers as well. *L. rohita* (Indian Major Carp) and *M. vittaus* (Bagridae catfish) are two of the most important commercial and cultivable freshwater fish species in India (Kumari et al., 2022; Mehar et al., 2022; Mawa et al., 2022), and the farmers badly need low-cost feed to grow them in rural areas. This study establishes that whey is a cheap and viable inoculum to ferment animal protein blends. For carnivorous fish, it produces a superior quality of feed ingredients as compared to a pure culture of *Lactobacillus acidophilus*.

## Conclusion

It is established from this study that WFAPB-supplemented feed results in increased availability and accumulation of the EEA and NAA in the plasma of stomach-less carp fish *L. rohita* and stomach-bearing carnivorous fish *M. vittatus*. However, such feed results in excess accumulation of many EEA and NAA in plasma, and only a small percent of these AA can be converted into crude protein and deposition of the bound AA in the muscle of these two fish. Using quadratic regression followed by classical optimization reveals that the optimum level of FM replacement by WFAPB ranges from 57.70 to 100% and 60.05 to 100% for AA absorption in plasma of *L.rohita* and *M. vittatus*, respectively. Whereas, the optimal FM replacement level varies between 0 and 16.02% for four growth parameters and 0 to 26 % for fifteen AA deposition parameters of *L.rohita*. The optimal FM replacement level for growth and AA deposition parameters of *M. vittatus* ranges between 36.64 to 37.58% and 37.5 to 42.96%, respectively. Applying two-phase fuzzy goal programming on four growth parameters (WG, SGR, FCR, and PER) and fifteen amino acid deposition parameters, it was revealed that FM that could be replaced for growth and amino acid deposition, respectively was only 7.63% and 12.23% for *L.rohita* and 36.79% and 40.02% for *M. vittatus*. It is, therefore, concluded that WFAPB is a promising FM replacer in the formulation of feed for stomach-bearing carnivorous fish like *M. vittatus*, and 36–40% replacement of FM by the WFAPB provides a successful outcome of growth and AA deposition in muscle. At this FM replacement level, it is unlikely to develop hyperaminoacidic conditions in plasma. In the present socio-economic scenario of the Indian sub-continent, the use of fish offal and slaughterhouse blood as alternative protein sources and whey as inoculum to ferment these protein sources would be economically viable for marginal fish farmers to prepare their own homemade fish feed.

## Data Availability

The datasets analyzed during the current study are available on request from A.K. and A.S.

## Appendix for “Recycling of animal protein wastes in the formulation of feed for *Labeo rohita* and *Mystus vittatus*—a comparative evaluation"

### Appendix A: Correlation coefficients in different scenarios

**Table 10:** Correlation coefficients for PP-AA absorption in blood for *L. rohita* and *M. vittatus*

	WFAPB	Arg	His	Iso	Leu	Lys	Met	Phe	Val	Asp	Cys	Glu	Gly	Pro	Ser	Tyr
*L. rohita*																
WFAPB	1	.899*	0.795	.901*	.949*	.941*	0.575	.896*	.917*	.967**	.903*	0.797	.998**	0.709	0.426	.950*
Arg	.899*	1	.946*	0.688	0.718	0.716	0.776	.943*	.974**	0.816	0.786	0.875	.892*	0.701	0.661	.936*
His	0.795	.946*	1	0.552	0.576	0.585	.937*	.965**	.959**	0.689	0.699	.952*	0.776	0.683	0.855	.913*
Iso	.901*	0.688	0.552	1	.953*	.984**	0.303	0.742	0.761	.979**	.967**	0.668	.920*	0.369	0.244	0.741
Leu	.949*	0.718	0.576	.953*	1	.990**	0.334	0.742	0.759	.964**	.885*	0.629	.954*	0.604	0.183	0.829
Lys	.941*	0.716	0.585	.984**	.990**	1	0.346	0.763	0.777	.982**	.935*	0.671	.951*	0.513	0.236	0.809
Met	0.575	0.776	.937*	0.303	0.334	0.346	1	0.857	0.815	0.443	0.483	.899*	0.544	0.613	.950*	0.777
Phe	.896*	.943*	.965**	0.742	0.742	0.763	0.857	1	.992**	0.839	0.853	.979**	.886*	0.642	0.783	.949*
Valin	.917*	.974**	.959**	0.761	0.759	0.777	0.815	.992**	1	0.863	0.866	.953*	.911*	0.641	0.735	.949*
Asp	.967**	0.816	0.689	.979**	.964**	.982**	0.443	0.839	0.863	1	.971**	0.754	.979**	0.513	0.350	0.852
Cys	.903*	0.786	0.699	.967**	.885*	.935*	0.483	0.853	0.866	.971**	1	0.812	.921*	0.352	0.463	0.789
Glu	0.797	0.875	.952*	0.668	0.629	0.671	.899*	.979**	.953*	0.754	0.812	1	0.787	0.525	0.878	0.874
Gly	.998**	.892*	0.776	.920*	.954*	.951*	0.544	.886*	.911*	.979**	.921*	0.787	1	0.668	0.406	.931*
Pro	0.709	0.701	0.683	0.369	0.604	0.513	0.613	0.642	0.641	0.513	0.352	0.525	0.668	1	0.336	0.833
Ser	0.426	0.661	0.855	0.244	0.183	0.236	.950*	0.783	0.735	0.350	0.463	0.878	0.406	0.336	1	0.611
Tyr	.950*	.936*	.913*	0.741	0.829	0.809	0.777	.949*	.949*	0.852	0.789	0.874	.931*	0.833	0.611	1
*M. vittatus*																
WFAPB	1	0.847	.896*	.882*	.987**	.966**	.901*	.969**	.975**	.976**	.926*	.991**	0.567	.962**	0.291	0.681
Arg	0.847	1	.963**	.987**	0.878	0.689	.937*	.928*	0.811	0.717	.908*	0.788	.917*	.914*	0.720	.950*
His	.896*	.963**	1	.959**	.886*	0.766	.995**	.959*	0.871	0.784	.966**	0.831	0.811	.900*	0.625	0.874
Iso	.882*	.987**	.959**	1	.916*	0.733	.930*	.962**	0.817	0.764	.948*	0.828	0.866	.917*	0.701	.946*
Leu	.987**	0.878	.886*	.916*	1	.934*	0.876	.972**	.944*	.952*	.917*	.980**	0.629	.976**	0.361	0.741
Lys	.966**	0.689	0.766	0.733	.934*	1	0.787	0.876	.965**	.998**	0.816	.986**	0.350	.898*	0.034	0.471
Met	.901*	.937*	.995**	.930*	0.876	0.787	1	.948*	.891*	0.799	.956*	0.837	0.767	.894*	0.563	0.825
Phe	.969**	.928*	.959*	.962**	.972**	0.876	.948*	1	.913*	.898*	.984**	.932*	0.707	.944*	0.510	0.827
Val	.975**	0.811	0.871	0.817	.944*	.965**	.891*	.913*	1	.963**	0.857	.972**	0.534	.957*	0.189	0.598
Asp	.976**	0.717	0.784	0.764	.952*	.998**	0.799	.898*	.963**	1	0.839	.993**	0.386	.912*	0.083	0.514
Cys	.926*	.908*	.966**	.948*	.917*	0.816	.956*	.984**	0.857	0.839	1	0.871	0.699	0.875	0.569	0.834
Glu	.991**	0.788	0.831	0.828	.980**	.986**	0.837	.932*	.972**	.993**	0.871	1	0.487	.952*	0.183	0.603
Gly	0.567	.917*	0.811	0.866	0.629	0.350	0.767	0.707	0.534	0.386	0.699	0.487	1	0.710	.881*	.957*
Pro	.962**	.914*	.900*	.917*	.976**	.898*	.894*	.944*	.957*	.912*	0.875	.952*	0.710	1	0.385	0.761
Ser	0.291	0.720	0.625	0.701	0.361	0.034	0.563	0.510	0.189	0.083	0.569	0.183	.881*	0.385	1	.890*
Tyr	0.681	.950*	0.874	.946*	0.741	0.471	0.825	0.827	0.598	0.514	0.834	0.603	.957*	0.761	.890*	1

Notes: *. Correlation is significant at the 0.05 level (2-tailed)

**. Correlation is significant at the 0.01 level (2-tailed)

**Table 11:** Correlation coefficients of growth parameters

	*L. rohita*					*M. vittatus*				
	WFAPB	WG	SGR	FCR	PER	WFAPB	WG	SGR	FCR	PER
WFAPB	1	−.906*	−.957*	.948*	−.967**	1	$-$0.656	$-$0.567	0.629	$-$0.618
WG	−.906*	1	.982**	−.992**	.936*	$-$0.656	1	.979**	−.997**	.994**
SGR	−.957*	.982**	1	−.990**	.984**	$-$0.567	.979**	1	−.967**	.966**
FCR	.948*	−.992**	−.990**	1	−.959**	0.629	−.997**	−.967**	1	−.999**
PER	−.967**	.936*	.984**	−.959**	1	$-$0.618	.994**	.966**	−.999**	1

Notes: *. Correlation is significant at the 0.05 level (2-tailed)

**. Correlation is significant at the 0.01 level (2-tailed)

**Table 12:** Correlation coefficient for AA deposition in muscle of *L. rohita* and *M. vittatus*

	*L. rohita*															
	WFBFS	Arg	His	Iso	Leu	Lys	Met	Phe	Val	Asp	Cys	Glu	Gly	Pro	Ser	Tyr
WFBFS	1	$-$0.847	−.901*	−.882*	−.895*	−.902*	$-$0.788	−.916*	$-$0.856	−.901*	−.914*	−.913*	−.916*	−.954*	−.945*	−.889*
Arg	$-$0.847	1	.951*	.993**	.979**	.962**	0.821	.971**	.936*	.978**	0.830	.987**	.965**	.882*	.919*	0.855
His	−.901*	.951*	1	.978**	.974**	.930*	.946*	.958*	.995**	.977**	.933*	.972**	.952*	.975**	.964**	.887*
Iso	−.882*	.993**	.978**	1	.995**	.974**	0.876	.987**	.965**	.995**	.891*	.996**	.982**	.925*	.957*	.899*
Leu	−.895*	.979**	.974**	.995**	1	.987**	.884*	.996**	.961**	1.000**	.922*	.996**	.994**	.930*	.977**	.938*
Lys	−.902*	.962**	.930*	.974**	.987**	1	0.813	.996**	.906*	.986**	.905*	.985**	.998**	.893*	.970**	.956*
Met	$-$0.788	0.821	.946*	0.876	.884*	0.813	1	0.853	.968**	.889*	.933*	0.859	0.848	.930*	.902*	0.841
Phe	−.916*	.971**	.958*	.987**	.996**	.996**	0.853	1	.937*	.996**	.922*	.995**	1.000**	.924*	.981**	.952*
Val	$-$0.856	.936*	.995**	.965**	.961**	.906*	.968**	.937*	1	.964**	.925*	.952*	.931*	.959**	.946*	0.870
Asp	−.901*	.978**	.977**	.995**	1.000**	.986**	.889*	.996**	.964**	1	.927*	.996**	.994**	.936*	.980**	.939*
Cys	−.914*	0.830	.933*	.891*	.922*	.905*	.933*	.922*	.925*	.927*	1	.903*	.926*	.956*	.979**	.963**
Glu	−.913*	.987**	.972**	.996**	.996**	.985**	0.859	.995**	.952*	.996**	.903*	1	.992**	.933*	.970**	.918*
Gly	−.916*	.965**	.952*	.982**	.994**	.998**	0.848	1.000**	.931*	.994**	.926*	.992**	1	.921*	.983**	.959**
Pro	−.954*	.882*	.975**	.925*	.930*	.893*	.930*	.924*	.959**	.936*	.956*	.933*	.921*	1	.963**	.884*
Ser	−.945*	.919*	.964**	.957*	.977**	.970**	.902*	.981**	.946*	.980**	.979**	.970**	.983**	.963**	1	.974**
Tyr	−.889*	0.855	.887*	.899*	.938*	.956*	0.841	.952*	0.870	.939*	.963**	.918*	.959**	.884*	.974**	1
	*M. Vittatus*															
	WFAPB	Arg	His	Iso	Leu	Lys	Met	Phe	Val	Asp	Cys	Glu	Gly	Ser	Tyr	
WFAPB	1	$-$0.378	$-$0.466	$-$0.495	$-$0.477	$-$0.488	$-$0.528	$-$0.331	$-$0.535	$-$0.361	$-$0.627	$-$0.536	$-$0.455	$-$0.646	$-$0.501	$-$0.423
Arg	$-$0.378	1	.972**	.941*	.958*	.982**	.939*	.978**	.979**	.972**	.883*	.957*	.992**	0.844	.975**	.960**
His	$-$0.466	.972**	1	0.870	.921*	.952*	.907*	.984**	.957*	.965**	0.821	.926*	.991**	0.777	.992**	.888*
Iso	$-$0.495	.941*	0.870	1	.967**	.974**	.963**	0.866	.972**	.897*	.983**	.975**	.925*	.969**	.903*	.995**
Leu	$-$0.477	.958*	.921*	.967**	1	.992**	.997**	.933*	.985**	.969**	.935*	.997**	.962**	.921*	.959**	.978**
Lys	$-$0.488	.982**	.952*	.974**	.992**	1	.985**	.952*	.998**	.970**	.940*	.995**	.983**	.917*	.976**	.982**
Met	$-$0.528	.939*	.907*	.963**	.997**	.985**	1	.913*	.980**	.955*	.943*	.997**	.948*	.934*	.951*	.969**
Phe	$-$0.331	.978**	.984**	0.866	.933*	.952*	.913*	1	.945*	.989**	0.793	.924*	.986**	0.750	.982**	.899*
Val	$-$0.535	.979**	.957*	.972**	.985**	.998**	.980**	.945*	1	.960**	.947*	.992**	.984**	.922*	.978**	.975**
Asp	$-$0.361	.972**	.965**	.897*	.969**	.970**	.955*	.989**	.960**	1	0.833	.957*	.981**	0.803	.982**	.928*
Cys	$-$0.627	.883*	0.821	.983**	.935*	.940*	.943*	0.793	.947*	0.833	1	.955*	0.877	.996**	0.863	.961**
Glu	$-$0.536	.957*	.926*	.975**	.997**	.995**	.997**	.924*	.992**	.957*	.955*	1	.964**	.941*	.962**	.979**
Gly	$-$0.455	.992**	.991**	.925*	.962**	.983**	.948*	.986**	.984**	.981**	0.877	.964**	1	0.840	.995**	.941*
pro	$-$0.646	0.844	0.777	.969**	.921*	.917*	.934*	0.750	.922*	0.803	.996**	.941*	0.840	1	0.829	.943*
Ser	$-$0.501	.975**	.992**	.903*	.959**	.976**	.951*	.982**	.978**	.982**	0.863	.962**	.995**	0.829	1	.919*
Tyr	$-$0.423	.960**	.888*	.995**	.978**	.982**	.969**	.899*	.975**	.928*	.961**	.979**	.941*	.943*	.919*	1

Notes: *. Correlation is significant at the 0.05 level (2-tailed)

**. Correlation is significant at the 0.01 level (2-tailed)

### Appendix C: Solution procedure for two-phase fuzzy goal programming

In this section, we explain the solution procedure for two-phase fuzzy goal programming method with explanation (Wu et al., 2015; Ali et al., 2020). First, we introduce the following definition in the context of multi-objective optimization problem:

#### Definition 1

Multiple objective optimization problems can be represented as follows:

$$\begin{aligned} \left\{ \begin{array}{ll} \text {max} &{} (f_1(x), f_2(x),\dots , f_k(x) )\\ \text {min} &{} (g_1(x), g_2(x),\dots , g_r(x) )\\ s.t. &{} x\in X=\{x\mid z_t(x) \le 0, t=1, \dots ,m\} \end{array} \right. \end{aligned}$$

where $x = (x_1, x_2,\dots ,x_n)$ are the decision variables; $f_i(x), (i = 1,\dots ,k)$ are maximization type objective functions; $g_j(x), (j = 1,\dots ,r)$ are minimization type objective function; $z_t(x), (t = 1,\dots ,m)$ are set of constraints.

In the context of our problem, we have only on variable with minimization objective (FCR), and rest of the objectives are of maximization type. We have only constraints, the optimal FM, denoted by *x*, and it should satisfy the range, $0\le x \le 100$.

#### Definition 2

A decision plan $x^0\in X$ is said to be a Pareto optimal solution to the multiple objective optimization problems if there does not exist another $y\in X$, such that $f_k(y)\le f_k (x^0)$ for all *k* and $f_s(y)< f_s (x^0)$ for at least one *s*.

#### Definition 3

A decision plan $x^0\in X$ is said to be a fuzzy-efficient solution if there does not exist another $y\in X$, such that $\mu _k(f_k(y))\ge \mu _k(f_k(y))$ is for all k and $\mu _s(f_s(y))\ge \mu _s(f_s(y))$ for at least one s.

For multiple objective optimization problem, optimal dose for all the objectives might be achieved simultaneously. Therefore, researchers are seeking **Pareto optimal solution**, which prevents the improved solution for an individual objective from worsening one or more other objectives. However, Pareto-optimally does not ensure fuzzy-efficiency solution. Therefore, we employed the two-phase method (Wu et al., 2015) to obtain solution:

**Step 1:** *Determine the positive ideal solution (*${f_t}^{min}$*) and the negative ideal solution (*${f_t}^{max}$*) for each objective function; by each objective function while ignoring the other objective function.*

For instance, we present the optimal dose for each objectives in the following Table 13 for rahu fish.

As we discussed earlier, negative optimal dose or optimal dose higher than 100%, replacement does not make any feasible solution. Therefore, we made the modified optimal replacement as presented in last row. Note that Table 13 is diagonally dominated. For instance, the first column represents the values of other variables at the optimal dose at which the Arg. reach the maximum value (26.0211%). Similarly, if we look at Cys. through the classical optimization it reach it maximum at $-$37.6316%. Therefore, we consider 0% when we made further computation.

**Step 2:** *Construct linear membership functions featuring both the continuously increasing property of the maximization objective function (**t**) and decreasing property of the minimization objective function (**r**) as follows:*

$$\begin{aligned} \mu _t(f_t) = \left\{ \begin{array}{ll} \frac{f_t-{f_t}^{min}}{{f_t}^{max}-{f_t}^{min}} &{} \text {if}\ {f_t}^{min}<f_t < {f_t}^{max}\\ 0 &{} \text {if}\ f_t\le {f_t}^{min} \end{array} \right. \end{aligned}$$

$$\begin{aligned} \mu _t(f_t) = \left\{ \begin{array}{ll} \frac{{f_t}^{max}(S)-f_t}{{f_t}^{max}-{f_t}^{min}} &{} \text {if}\ {f_t}^{min}<f_t < {f_t}^{max}\\ 0 &{} \text {if}\ f_t\le {f_t}^{max} \end{array} \right. \end{aligned}$$

where the possible range for the r-th objective $({f_t}^{min}, {f_t}^{max})$ is constructed from the optimal solution of the problem by incorporating only one objective function while ignoring the other objective function.

For instance, we made the following membership function for *Arg* and *PER* in the context of *L. rohita* fish:

$$\begin{aligned} \mu _t(f_{arg}) = \left\{ \begin{array}{ll} \frac{f_{arg(S)}-4.4048}{4.4466-4.4048} &{} \text {if}\ 4.4048<f_{arg}(S) < 4.4466\\ 0 &{} \text {if}\ f_t\le 4.4048 \end{array} \right. \end{aligned}$$

$$\begin{aligned} \mu _t(f_{PER}) = \left\{ \begin{array}{ll} \frac{1.3517-f_{PER}(S)}{1.3517-1.3490} &{} \text {if}\ 1.3490<f_{PER}(S) < 1.3517\\ 0 &{} \text {if}\ f_{PER}(S)\le 1.3517 \end{array} \right. \end{aligned}$$

**Step 3:** *Under the two-phase approach, one needs to determine the optimal solution by solving the following optimization problem in Phase I:*

A.1 $$\begin{aligned} \max \quad Z_1(x)&= \lambda \end{aligned}$$

A.2 $$\begin{aligned} s.t. \quad&\mu _k(f_k)\ge \lambda , \forall k\end{aligned}$$

A.3 $$\begin{aligned} \quad&0\le x\le 100, \quad \lambda \ge 0 \end{aligned}$$

**Step 4:** *The two-phase approach provides the flexibility to reach optimal goals by relaxing this constraint in Phase I. In Phase II, we need to determine the following optimization problem to obtain the optimal dose.*

A.4 $$\begin{aligned} \max \quad Z_2&= \lambda \end{aligned}$$

A.5 $$\begin{aligned} s.t. \quad&\mu _k(f_k) -\rho _k \ge \lambda ^{*},\quad \lambda ^{*}\ge 0,\quad \rho _k \ge 0, \forall k\end{aligned}$$

A.6 $$\begin{aligned} \quad&0\le x\le 100, \quad \lambda \ge 0 \end{aligned}$$

*where* $\lambda ^{*}$ *is the optimal value of* $\lambda$ *obtained in Phase I. It is to be noted that if* $\rho _k=0$, *then there are no solutions with better efficiency for the model under Phase I(*Wu et al. (2015); Ali et al. (2020)*. If* $\rho _k>0$ *for some* *k**, the solution obtained from Phase II is more efficient compared to the solution obtained from Phase I, and the decision maker would be able to obtain information for achieving subjective goals.*

If our objective is to maximize only WG, the optimal FM replacement level if 16.09%. However, if we focus on the forth growth parameters the optimal FM replacement by using FGM, it will be 7.63%. The rational behind the findings is that, when we consider SGR and PER into the account, their FM replacement become 0%, therefore, the combine level decrease to ensure the Parato optimal solution. Proceeding in the similar way, we obtain the optimal replacement as 12.23% when nineteen parameters are considered.

**Table 13:** Values of different parameters in different optimal doses for *L. rohita*

Obj	Max	Max	Max	Max	Max	Max	Max	Max	Max	Max	Max	Max	Max	Max	Max	Max	Max	Min	Max
	Arg	Hist	Isod	Leu	Lys	Metf	Phe	val	Asp	Cys	Glu	Gly	Pro	Ser	Try	WG	SGR	FCR	PER
Arg	**4**.**4466**	4.4344	4.4456	4.4441	4.4417	4.4346	4.4397	4.4426	4.4430	4.4049	4.4414	4.4386	4.4049	4.4049	4.4055	4.4405	4.4049	4.4181	4.4049
Hist	2.2766	**2**.**2805**	2.2786	2.2793	2.2800	2.2805	2.2802	2.2798	2.2797	2.2777	2.2800	2.2803	2.2777	2.2777	2.2778	2.2802	2.2777	2.2794	2.2777
Isod	2.6403	2.6372	**2**.**6409**	2.6407	2.6400	2.6373	2.6393	2.6403	2.6405	2.6229	2.6399	2.6389	2.6229	2.6229	2.6232	2.6396	2.6229	2.6295	2.6229
Leu	4.9526	4.9519	4.9542	4.9544	4.9541	4.9519	4.9537	4.9543	4.9544	4.9377	4.9541	4.9533	4.9377	4.9377	4.9381	4.9539	4.9377	4.9445	4.9377
Lys	5.4429	5.4456	5.4457	5.4465	5.4468	5.4456	5.4467	5.4468	5.4468	5.4326	5.4468	5.4465	5.4326	5.4326	5.4329	5.4468	5.4326	5.4391	5.4326
Met	1.0263	1.0290	1.0277	1.0282	1.0286	1.0290	1.0288	1.0285	1.0284	1.0270	1.0287	1.0289	1.0270	1.0270	1.0271	1.0288	1.0270	1.0282	1.0270
Phe	4.8913	4.8960	4.8946	4.8957	4.8964	4.8960	4.8966	4.8963	4.8962	4.8854	4.8965	4.8965	4.8854	4.8854	4.8857	4.8966	4.8854	4.8910	4.8854
val	0.7404	0.7408	0.7410	0.7412	0.7412	0.7408	0.7412	0.7413	0.7413	0.7369	0.7412	0.7411	0.7369	0.7369	0.7370	0.7412	0.7369	0.7388	0.7369
Asp	6.5208	6.5219	6.5238	6.5245	6.5245	6.5220	6.5240	6.5246	6.5246	6.5026	6.5244	6.5236	6.5026	6.5026	6.5031	6.5242	6.5026	6.5120	6.5026
Cys	0.2858	0.2949	0.2887	0.2902	0.2919	0.2949	0.2929	0.2913	0.2910	0.3009	0.2920	0.2934	0.3009	0.3009	0.3008	0.2925	0.3009	0.2988	0.3009
Glu	6.7930	6.7965	6.7963	6.7973	6.7978	6.7965	6.7977	6.7977	6.7976	6.7820	6.7978	6.7975	6.7820	6.7820	6.7824	6.7977	6.7820	6.7893	6.7820
Gly	6.1006	6.1067	6.1044	6.1058	6.1067	6.1067	6.1070	6.1065	6.1063	6.0966	6.1068	6.1070	6.0966	6.0966	6.0969	6.1069	6.0966	6.1020	6.0966
Pro	3.2589	3.2778	3.2648	3.2679	3.2713	3.2777	3.2734	3.2702	3.2696	3.2910	3.2717	3.2745	3.2910	3.2910	3.2908	3.2727	3.2910	3.2863	3.2910
Ser	3.1536	3.1672	3.1584	3.1608	3.1632	3.1671	3.1646	3.1624	3.1620	3.1723	3.1634	3.1652	3.1723	3.1723	3.1722	3.1641	3.1723	3.1710	3.1723
Try	1.5315	1.5394	1.5344	1.5358	1.5372	1.5393	1.5380	1.5368	1.5365	1.5414	1.5373	1.5383	1.5414	1.5414	1.5414	1.5377	1.5414	1.5412	1.5414
WGR	291.554	291.979	291.888	291.998	292.059	291.983	292.062	292.046	292.036	290.756	292.061	292.054	290.756	290.756	290.788	292.064	290.756	291.389	290.757
SGR	0.6469	0.6501	0.6480	0.6485	0.6491	0.6500	0.6494	0.6489	0.6488	0.6517	0.6491	0.6496	0.6517	0.6517	0.6517	0.6493	0.6517	0.6512	0.6517
FCR	1.3517	1.3493	1.3507	1.3503	1.3499	1.3493	1.3497	1.3500	1.3501	1.3491	1.3499	1.3496	1.3491	1.3491	1.3491	1.3498	1.3491	1.3490	1.3491
PER	2.4487	2.4580	2.4515	2.4530	2.4547	2.4579	2.4558	2.4542	2.4539	2.4651	2.4549	2.4563	2.4651	2.4651	2.4650	2.4554	2.4651	2.4625	2.4651
AO	26.0211	11.9330	21.8966	19.6198	17.0468	12.0350	15.4158	17.9197	18.3871	$-$37.6316	16.7857	14.6029	$-$55.5556	$-$4.4418	0.2013	16.0198	$-$14.2794	4.5088	$-$124.2170
MO	26.0211	11.9330	21.8966	19.6198	17.0468	12.0350	15.4158	17.9197	18.3871	0.0000	16.7857	14.6029	0.0000	0.0000	0.2013	16.0198	0.0000	4.5088	0.0000

AO-actual optimal; and MO- modified optimal

## References

[CR1] Achmad, H., Chaklader, M.R., Fotedar, R., Foysal, M.J., 2023. From waste to feed: Microbial fermented abalone waste improves the digestibility, gut health, and immunity in marron, *Cherax cainii*. Fish & Shellfish Immunology , 108748. 10.1016/j.fsi.2023.108748 37087026

[CR2] Ali, S., Saha, S., Kaviraj, A., 2020. Fermented mulberry leaf meal as fishmeal replacer in the formulation of feed for carp *Labeo rohita* and catfish *Heteropneustes* fossilis-optimization by mathematical programming. Tropical animal health and production 52, 839–849. 10.1007/s11250-019-02075-x 31586318

[CR3] Ambardekar, A.A., Reigh, R.C., Williams, M.B., 2009. Absorption of amino acids from intact dietary proteins and purified amino acid supplements follows different time-courses in channel catfish (ictalurus punctatus). Aquaculture 291, 179–187.

[CR4] Bai, X., Jia, J., Kang, Q., Fu, Y., Zhou, Y., Zhong, Y., Zhang, C., Li, M., 2021. Integrated metabolomics and lipidomics analysis reveal remodeling of lipid metabolism and amino acid metabolism in glucagon receptor–deficient zebrafish. Frontiers in Cell and Developmental Biology 8, 605979. 10.3389/fcell.2020.605979 PMC7841139 33520988

[CR5] Boirie, Y., Dangin, M., Gachon, P., Vasson, M.P., Maubois, J.L., Beaufrère, B., 1997. Slow and fast dietary proteins differently modulate postprandial protein accretion. Proceedings of the national academy of sciences 94, 14930–14935. 10.1073/pnas.94.26.14930 PMC25140 9405716

[CR6] Brafield, A., 1985. Laboratory studies of energy budgets. Fish energetics: new perspectives , 257–281.

[CR7] Brezas, A., Hardy, R.W., 2020. Improved performance of a rainbow trout selected strain is associated with protein digestion rates and synchronization of amino acid absorption. Scientific Reports 10, 1–12. 10.1038/s41598-020-61360-0 PMC7069933 32170085

[CR8] Carter, C.G., Mente, E., Barnes, R.K., Nengas, I., 2012. Protein synthesis in gilthead sea bream: response to partial fishmeal replacement. British journal of nutrition 108, 2190–2197. 10.1017/S0007114512000426 22414773

[CR9] Chakrabarti, P., Ghosh, S.K., 2014. A comparative study of the histology and microanatomy of the stomach in *Mystus vittatus* (Bloch), *Lizaparsia* (Hamilton) and *Oreochromis mossambicus* (Peters). Journal of Microscopy and Ultrastructure 2, 245–250.

[CR10] Chowdhury, M.W., Nabi, M.N., Arefin, M.A., Rashid, F., Islam, M.T., Gudimetla, P., Muyeen, S., 2022. Recycling slaughterhouse wastes into potential energy and hydrogen sources: An approach for the future sustainable energy. Bioresource Technology Reports , 101133.

[CR11] Dalsgaard, J., Ekmann, K.S., Jensen, M.D., Pedersen, P.B., 2023. Reducing phosphorus emissions from net cage fish farming by diet manipulation. Journal of Environmental Management 334, 117445. 10.1016/j.jenvman.2023.117445 36774900

[CR12] Das, B., Sarkar, S., Sarkar, A., Bhattacharjee, S., Bhattacharjee, C., 2016. Recovery of whey proteins and lactose from dairy waste: A step towards green waste management. Process Safety and Environmental Protection 101, 27–33.

[CR13] Debnath, D., Pal, A., Sahu, N., Yengkokpam, S., Baruah, K., Choudhury, D., Venkateshwarlu, G., 2007. Digestive enzymes and metabolic profile of *Labeo rohita* fingerlings fed diets with different crude protein levels. Comparative Biochemistry and Physiology Part B: Biochemistry and Molecular Biology 146, 107–114. 10.1016/j.cbpb.2006.09.008 17112756

[CR14] Dileep, N., Pradhan, C., Peter, N., Kaippilly, D., Sashidharan, A., Sankar, T., 2021. Nutritive value of guar and copra meal after fermentation with yeast saccharomyces cerevisiae in the diet of nile tilapia, *Oreochromis niloticus*. Tropical Animal Health and Production 53, 1–13. 10.1007/s11250-021-02855-4 34313860

[CR15] Espe, M., Lied, E., 1994. Do atlantic salmon (*Salmo salar*) utilize mixtures of free amino acids to the same extent as intact protein sources for muscle protein synthesis? Comparative Biochemistry and Physiology Part A: Physiology 107, 249–254.

[CR16] Gao, S., Sun, P., Ren, H., Chen, J., Shen, Y., Wang, Z., Huang, Y., Chen, W., 2023. Effects of dietary phosphorus deficiency on the growth performance, hepatic lipid metabolism, and antioxidant capacity of yellow river carp Cyprinus carpio *haematopterus*. Journal of Aquatic Animal Health . 10.1002/aah.10177 36861820

[CR17] Giromini, C., Ottoboni, M., Tretola, M., Marchis, D., Gottardo, D., Caprarulo, V., Baldi, A., Pinotti, L., 2017. Nutritional evaluation of former food products (ex-food) intended for pig nutrition. Food additives & contaminants: Part A 34, 1436–1445. 10.1080/19440049.2017.1306884 28397545

[CR18] Goncalves, O., Castro, L.F.C., Smolka, A.J., Fontainhas, A., Wilson, J.M., 2016. The gastric phenotype in the cypriniform loaches: A case of reinvention? PloS one 11, e0163696. 10.1371/journal.pone.0163696 PMC5082673 27783698

[CR19] Hardy, R.W., 2010. Utilization of plant proteins in fish diets: effects of global demand and supplies of fishmeal. Aquaculture research 41, 770–776.

[CR20] Helrich, K., 1990. Official methods of analysis of the Association of Official Analytical Chemists. BOOK, Association of official analytical chemists.

[CR21] Hua, K., Cobcroft, J.M., Cole, A., Condon, K., Jerry, D.R., Mangott, A., Praeger, C., Vucko, M.J., Zeng, C., Zenger, K., et al., 2019. The future of aquatic protein: implications for protein sources in aquaculture diets. One Earth 1, 316–329.

[CR22] Jobling, M., 2012. National research council (nrc): Nutrient requirements of fish and shrimp: The national academies press, washington, dc, 2011, 376+ xvi pp,£ 128 (hardback), isbn: 978-0-309-16338-5.

[CR23] Katersky, R.S., Carter, C.G., 2010. The effect of temperature on post-prandial protein synthesis in juvenile barramundi, lates calcarifer. Comparative Biochemistry and Physiology Part A: Molecular & Integrative Physiology 156, 529–536. 10.1016/j.cbpa.2010.04.009 20406695

[CR24] Kaushik, S.J., Seiliez, I., 2010. Protein and amino acid nutrition and metabolism in fish: current knowledge and future needs. Aquaculture research 41, 322–332.

[CR25] Khieokhajonkhet, A., Aeksiri, N., Rojtinnakorn, J., Van Doan, H., Kaneko, G., 2022. Sacha inchi meal as a fish-meal replacer in red hybrid tilapia (*Oreochromis niloticus*$$\times $$*O. mossambicus*) feeds: effects on dietary digestibility, growth metrics, hematology, and liver and intestinal histology. Aquaculture International 30, 677–698.

[CR26] Kumari, K., Singh, A., Swamy, S., Singhar, R.S., Thakur, S., 2022. Use of enzymatic biomarkers of *Labeo rohita* to study the effect of polybrominated diphenyl ether (bde-209) via dietary exposure in laboratory conditions. Environmental Monitoring and Assessment 194, 499. 10.1007/s10661-022-09963-0 35695941

[CR27] Kwanyuen, P., Burton, J.W., 2010. A modified amino acid analysis using pitc derivatization for soybeans with accurate determination of cysteine and half-cystine. Journal of the American Oil Chemists’ Society 87, 127–132.

[CR28] Langi, S., Panana, E., Alloo, C., Van Stappen, G., Meeus, W., 2023. Replacement of fishmeal using poultry-based protein sources in 886 feeds for pikeperch (*Sander lucioperca, Linnaeus*, 1758) during grow out phase. Aquaculture International 31, 65–80.

[CR29] Larsen, B.K., Dalsgaard, J., Pedersen, P.B., 2012. Effects of plant proteins on postprandial, free plasma amino acid concentrations in rainbow trout (*Oncorhynchus mykiss*). Aquaculture 326, 90–98.

[CR30] Li, P., Mai, K., Trushenski, J., Wu, G., 2009. New developments in fish amino acid nutrition: towards functional and environmentally oriented aquafeeds. Amino acids 37, 43–53. 10.1007/s00726-008-0171-1 18751871

[CR31] Matias, A.C., Ribeiro, L., Barata, M., Araújo, R.L., Pousão-Ferreira, P., 2023. Postprandial pattern of digestive enzymes and protein turnover in meagre (*Argyrosomus Regius*) juveniles. Comparative Biochemistry and Physiology Part B: Biochemistry and Molecular Biology , 110828. 10.1016/j.cbpb.2023.110828 36634814

[CR32] Mawa, Z., Hossain, M.Y., Hasan, M.R., Rahman, M.A., Tanjin, S., Ohtomi, J., 2022. Life history traits of mystus vittatus in the ganges river, bangladesh: recommendation for its sustainable management considering climate change. International Journal of Biometeorology 66, 927–943. 10.1007/s00484-022-02249-7 35211787

[CR33] Maynard, L., Loosil, J., Hintz, H., Warner, R., 1979. In cr zappa. Animal nutrition (7th ed., pp. 13–14). New York: McGraw-Hill .

[CR34] Mayta-Apaza, A.C., Rocha-Mendoza, D., García-Cano, I., Jiménez-Flores, R., 2022. Characterization and evaluation of proteolysis products during the fermentation of acid whey and fish waste and potential applications. ACS Food Science & Technology 2, 1442–1452. 10.1021/acsfoodscitech.2c00157 PMC9487912 36161074

[CR35] Mbokane, E.M., Mbokane, L.M., Fouche, C.H., 2022. The effect of fishmeal replacement with acid-fermented chicken silage on growth, digestive enzyme activity and histology of the intestine and liver of juvenile mozambique tilapia (*Oreochromis Mossambicus*). Aquaculture International 30, 2491–2512.

[CR36] McCarthy, I.D., Fuiman, L.A., 2011. Post-prandial changes in protein synthesis in red drum (*Sciaenops ocellatus*) larvae. Journal of Experimental Biology 214, 1821–1828. 10.1242/jeb.052753 21562168

[CR37] Mehar, M., Mekkawy, W., McDougall, C., Benzie, J.A., 2022. Preferences for rohu fish (*l. rohita*) traits of women and men from farming households in bangladesh and india. Aquaculture 547, 737480.

[CR38] Mente, E., Carter, C.G., Barnes, R.S., Vlahos, N., Nengas, I., 2021. Post-prandial amino acid changes in gilthead sea bream. Animals 11, 1889. 10.3390/ani11071889 PMC8300103 34201988

[CR39] Mente, E., Deguara, S., Santos, M.B., Houlihan, D., 2003. White muscle free amino acid concentrations following feeding a maize gluten dietary rotein in atlantic salmon (*Salmo salar l.*). Aquaculture 225, 133–147.

[CR40] Mishra, P.K., Parey, A., Saha, B., Samaddar, A., Chakraborty, S., Kaviraj, A., Nielsen, I., Saha, S., 2022. Production analysis of composite fish culture in drought prone areas of purulia: The implication of financial constraint. Aquaculture 548, 737629.

[CR41] Mugwanya, M., Dawood, M.A., Kimera, F., Sewilam, H., 2023. Replacement of fish meal with fermented plant proteins in the aquafeed industry: A systematic review and meta-analysis. Reviews in Aquaculture 15, 62–88.

[CR42] Naylor, R.L., Hardy, R.W., Buschmann, A.H., Bush, S.R., Cao, L., Klinger, D.H., Little, D.C., Lubchenco, J., Shumway, S.E., Troell, M., 2021. A 20-year retrospective review of global aquaculture. Nature 591, 551–563. 10.1038/s41586-021-03308-6 33762770

[CR43] Obirikorang, K.A., Amisah, S., Fialor, S.C., Skov, P.V., 2015. Local agro-industrial by-products with potential use in ghanaian aquaculture: a review. Aquaculture International 23, 403–425.

[CR44] Panserat, S., Kaushik, S., 2010. Regulation of gene expression by nutritional factors in fish. Aquaculture Research 41, 751–762.

[CR45] Rice, E.W., Bridgewater, L., Association, A.P.H., et al., 2012. Standard methods for the examination of water and wastewater. 10. American public health association Washington, DC.

[CR46] Samaddar, A., Kaviraj, A., 2014. Processing of fish offal waste through fermentation utilizing whey as inoculum. International Journal of Recycling of Organic Waste in Agriculture 3, 1–8.

[CR47] Samaddar, A., Kaviraj, A., 2015. Application of fermentation technology to use slaughterhouse blood as potential protein supplement in fish feed. Jordan Journal of Biological Sciences 8.

[CR48] Samaddar, A., Kaviraj, A., Nielsen, I.E., Saha, S., 2021. Replacement of fishmeal by fermented animal protein blend in the feed of *Mystus vittatus*: Analysis of optimality by programming and modeling, in: Proceedings of the Zoological Society, Springer. pp. 62–72.

[CR49] Samaddar, A., Kaviraj, A., Saha, S., 2015. Utilization of fermented animal by-product blend as fishmeal replacer in the diet of *Labeo rohita*. Aquaculture Reports 1, 28–36.

[CR50] Shabani, A., Boldaji, F., Dastar, B., Ghoorchi, T., Zerehdaran, S., 2018. Preparation of fish waste silage and its effect on the growth performance and meat quality of broiler chickens. Journal of the Science of Food and Agriculture 98, 4097–4103. 10.1002/jsfa.8926 29388212

[CR51] Shabani, A., Boldaji, F., Dastar, B., Ghoorchi, T., Zerehdaran, S., Ashayerizadeh, A., 2021. Evaluation of increasing concentrations of fish waste silage in diets on growth performance, gastrointestinal microbial population, and intestinal morphology of broiler chickens. Animal Feed Science and Technology 275, 114874.

[CR52] Shamsuzzaman, M.M., Mozumder, M.M.H., Mitu, S.J., Ahamad, A.F., Bhyuian, M.S., 2020. The economic contribution of fish and fish trade in bangladesh. Aquaculture and Fisheries 5, 174–181.

[CR53] Sharf, Y., Khan, M.A., 2023. Dietary leucine requirement of fingerling *Channa punctatus* (Bloch) based on growth, feed conversion and leucine retention efficiency, hematological parameters, antioxidant and intestinal enzyme activities. Amino Acids , 1–18. 10.1007/s00726-023-03240-1 36682022

[CR54] Siddik, M.A., Fotedar, R., Chaklader, M.R., Foysal, M.J., Nahar, A., Howieson, J., 2020. Fermented animal source protein as substitution of fishmeal on intestinal microbiota, immune-related cytokines and resistance to vibrio mimicus in freshwater crayfish (*Cherax cainii*). Frontiers in Physiology , 1635. 10.3389/fphys.2019.01635 PMC7005050 32082185

[CR55] Simtoe, A.P., Luvanga, S.A., Lugendo, B.R., 2022. Partial replacement of fishmeal with chaetomorpha algae improves feed utilization, survival, biochemical composition, and fatty acids profile of farmed shrimp *Penaeus monodon* post larvae. Aquaculture International , 1–14.

[CR56] Stone, F.E., Hardy, R.W., Shearer, K.D., Scott, T.M., 1989. Utilization of fish silage by rainbow trout (*Salmo gairdneri*). Aquaculture 76, 109–118.

[CR57] Tacon, A.G., Metian, M., 2015. Feed matters: satisfying the feed demand of aquaculture. Reviews in Fisheries Science & Aquaculture 23, 1–10.

[CR58] Tantikitti, C., March, B., 1995. Dynamics of plasma free amino acids in rainbow trout (*Oncorhynchus mykiss* under variety of dietary conditions. Fish Physiology and Biochemistry 14, 179–194. 10.1007/BF00004309 24197440

[CR59] Teles, A.O., Couto, A., Enes, P., Peres, H., 2020. Dietary protein requirements of fish–a meta-analysis. Reviews in Aquaculture 12, 1445–1477.

[CR60] Walton, M., Cowey, C., Coloso, R.M., Adron, J., 1986. Dietary requirements of rainbow trout for tryptophan, lysine and arginine determined by growth and biochemical measurements. Fish Physiology and Biochemistry 2, 161–169. 10.1007/BF02264084 24233178

[CR61] Wangkheirakpam, M., Mahanand, S., Majumdar, R., Sharma, S., Hidangmayum, D., Netam, S., 2019. Fish waste utilization with reference to fish protein hydrolisate-a review. Fish. Technol 56, 169–178.

[CR62] Wu, Y.K., Liu, C.C., Lur, Y.Y., 2015. Pareto-optimal solution for multiple objective linear programming problems with fuzzy goals. Fuzzy optimization and decision making 14, 43–55.

[CR63] Xu, D., He, G., Mai, K., Zhou, H., Xu, W., Song, F., 2016. Postprandial nutrient-sensing and metabolic responses after partial dietary fishmeal replacement by soyabean meal in turbot (*Scophthalmus maximus l.*). British Journal of Nutrition 115, 379–388. 10.1017/S0007114515004535 26586314

[CR64] Youssef, I.M., Saleh, E.S., Tawfeek, S.S., Abdel-Fadeel, A.A., Abdel-Razik, A.R.H., Abdel-Daim, A.S., 2023. Effect of spirulina platensis on growth, hematological, biochemical, and immunological parameters of nile tilapia (*Oreochromis niloticus*). Tropical Animal Health and Production 55, 275. 10.1007/s11250-023-03690-5 PMC10374666 37498411

[CR65] Yu, J., Yu, J., Chen, X., Zhou, X., Cai, Y., Cai, H., Yan, P., 2019. Effects of fermented protein feed on the growth performance of pond-raised crab. Aquaculture and Fisheries 4, 149–155.

[CR66] Zandona, E., Blažić, M., Režek Jambrak, A., 2021. Whey utilization: Sustainable uses and environmental approach. Food Technology and Biotechnology 59, 147–161. 10.17113/ftb.59.02.21.6968 PMC8284110 34316276

[CR67] Zar, J.H., 1999. Biostatistical analysis. Pearson Education India.

[CR68] Zhu, Z.H., Yang, Q.h., Tan, B.p., Zhou, X.Q., Dong, X.h., Chi, S.y., Liu, H.y., Zhang, S., 2021. Effects of replacing fishmeal with soybean protein concentrate (spc) on growth, blood biochemical indexes, non-specific immune enzyme activity, and nutrient apparent digestibility for juvenile *Litopenaeus vannamei*. Aquaculture International 29, 2535–2554.

